# A numerical study of spatio-temporal COVID-19 vaccine model via finite-difference operator-splitting and meshless techniques

**DOI:** 10.1038/s41598-023-38925-w

**Published:** 2023-07-26

**Authors:** Arshad A. Khan, Saif Ullah, Mohamed Altanji, Rohul Amin, Nadeem Haider, Ahmed Alshehri, Muhammad Bilal Riaz

**Affiliations:** 1 Department of Mathematics, University of Peshawar, Khyber Pakhtunkhwa, Pakistan; 2 Department of Mathematics College of Science, King Khalid University, Abha, 61413 Saudi Arabia; 3 Department of Mathematics, Faculty of Sciences, King Abdulaziz University, Jidda, 21589 Saudi Arabia; 4 Faculty of Applied physics and Mathematics, Gdansk University of Technology, Gdansk, Poland; 5 Department of Computer Science and Mathematics, Lebanese American University, Byblos, Lebanon; 6 Department of Mathematics, University of Management and Technology, Lahore, Pakistan

**Keywords:** Computational biology and bioinformatics, Computational models

## Abstract

In this paper, a new spatio-temporal model is formulated to study the spread of coronavirus infection (COVID-19) in a spatially heterogeneous environment with the impact of vaccination. Initially, a detailed qualitative analysis of the spatio-temporal model is presented. The existence, uniqueness, positivity, and boundedness of the model solution are investigated. Local asymptotical stability of the diffusive COVID-19 model at steady state is carried out using well-known criteria. Moreover, a suitable nonlinear Lyapunov functional is constructed for the global asymptotical stability of the spatio-temporal model. Further, the model is solved numerically based on uniform and non-uniform initial conditions. Two different numerical schemes named: finite difference operator-splitting and mesh-free operator-splitting based on multi-quadratic radial basis functions are implemented in the numerical study. The impact of diffusion as well as some pharmaceutical and non-pharmaceutical control measures, i.e., reducing an effective contact causing infection transmission, vaccination rate and vaccine waning rate on the disease dynamics is presented in a spatially heterogeneous environment. Furthermore, the impact of the  aforementioned interventions is investigated with and without diffusion on the incidence of disease. The simulation results conclude that the random motion of individuals has a significant impact on the disease dynamics and helps in setting a better control strategy for disease eradication.

## Introduction

COVID-19 is a viral disease caused by a variant of coronavirus named as severe acute respiratory syndrome coronavirus 2 (SARS-CoV-2). The disease transmission occurs between individuals through respiratory droplets generated when an infected person coughs or sneezes. The disease initially originated in December 2019 in China and has spread to the rest of the world rapidly. It caused public health burden and create an economic crisis around the globe. Despite the availability of various vaccines, it is still dangerous to public health. One of the major factors that rapid the transmission of COVID-19 viral disease is social contact and public gathering. Epidemiological models remain an effective tool to understand the geographical spreading pattern and the impact of those factors that are responsible for the spread and control of infection. In this regard, the most important thing is the numerical treatment of such models, since one might be interested in obtaining the projected information based on reported data. Using this information to make some useful control strategies that help in the eradication and control of infection. For this purpose, many researchers around the globe developed spatio-temporal epidemic models and discussed their approximate solutions via various iterative schemes to explore the dynamical behavior of infectious diseases. For instance, Imran et al.^1^ formulated a new compartmental model to investigate the dynamical behavior of influenza with the spatial and temporal effects. The authors solved the developed model by using a finite-difference scheme. Samsuzzoha et al.^2^ formulated a diffusive transmission model to analyze the dynamics of influenza with spatial and temporal effects. Jawaz et al.^3^ have investigated the transmission dynamics and control of HIV/AIDS using a mathematical spatio-temporal epidemic model. They proposed a non-standard finite difference numerical scheme for the numerical solution of the model due to its ability to preserve positivity. In the study conducted by Ahmed et al.^4^, an efficient numerical scheme is derived to demonstrate the graphical dynamics of the whooping cough transmission model. Following a similar approach, the works of Haider et al.^5,6^ and Asif et al.^7^ utilized operator-splitting-based finite difference and meshless procedures for solving spatio-temporal compartmental models. Asif et al.^8^ presented approximate solutions of two similar epidemic models via meshless and finite difference techniques. Ahmed et al.^9^ implemented a non-standard finite difference splitting scheme with structure-preserving property to investigate the numerical solution of a reaction–diffusion transmission model. Sokolovsky et al.^10^ studied both the numerical and analytic solutions of space-temporal compartmental models, analyzing multiple stages of a disease.

Motivated by the above literature, in the present investigation, the standard epidemic model is modified for the analysis of COVID-19 viral disease with the impact of vaccination in a spatially heterogeneous environment. Initially, the proposed model is analyzed qualitatively and solved numerically with two different procedures named: operator-splitting finite difference and operator-splitting meshless scheme based on multi-quadratic radial basis function, to compare the results mutually due to the non-availability of the exact solution. Furthermore, the impact of diffusion and important model parameters describing some pharmaceutical and non-pharmaceutical control measures are analyzed on the disease incidence. The rest of article is organized as: in “Model description” mathematical description of the proposed model is presented in detail. The qualitative analysis including boundedness and stability of model steady-states, is carried out in “Qualitative analysis”. The numerical schemes are discussed in  “Numerical methods”. Simulation and discussion are given in “Simulation and discussion”. Finally, “Conclusion” contains the concluding remarks.

## Model description

This section presents the formulation of the spatio-temporal COVID-19 model. A reaction–diffusion compartmental epidemic model is formulated to analyze the COVID-19 temporal and spatial dynamics. The total human population at time $t\ge 0$ and $x\in \Omega = [-2, 2]$ is spatially distributed as

$$\begin{aligned} \mathcal {N}(t) = \int _{\Omega }\bigg \{ S(x, t)+E(x, t)+I(x, t)+V(x, t)+R(x, t)\bigg \}dx, \end{aligned}$$

where *S*(*x*, *t*), *E*(*x*, *t*), *I*(*x*, *t*), *V*(*x*, *t*), and *R*(*x*, *t*) represent different categories of population based on their disease status. The compartment *S*(*x*, *t*) contains individuals that are at risk and can acquire the disease. Those who are in the latency period will be placed in exposed compartment *E*(*x*, *t*). The individuals that have fully developed symptoms are placed in *I*(*x*, *t*), while *V*(*x*, *t*) and *R*(*x*, *t*) are vaccinated and recovered compartments respectively. Furthermore, It is assumed that the populations *S*, *E*, *I*, *V*, and *R* are free to diffuse only in the *x* direction. The vaccinated individuals become susceptible after the vaccine-waning effect. The recovered individuals join the susceptible class on losing disease-acquired immunity. Based on the above assumptions the proposed phenomena is stated in the form of the system of PDEs represent by (1), which is the spatio-temporal extension of the vaccine model proposed in^11^ as follow:

1 $$\begin{aligned} \left. \begin{array}{ll} \dfrac{\partial S}{\partial t} = \alpha _S\dfrac{\partial ^2 S}{\partial x^2}+\Theta -\beta (I+\kappa E)\dfrac{S}{N}-(\psi _{\nu }+\mu )S+\eta _{\nu }V+\theta R, \\ \dfrac{\partial E}{\partial t} = \alpha _E\dfrac{\partial ^2 E}{\partial x^2}+\beta (I+\kappa E)\dfrac{S}{N}-(\mu +\gamma )E,\\ \dfrac{\partial I}{\partial t} = \alpha _I\dfrac{\partial ^2 I}{\partial x^2}+\gamma E - (\mu +\mu _{0}+\omega )I,\\ \dfrac{\partial V}{\partial t} = \alpha _V\dfrac{\partial ^2 V}{\partial x^2}+\psi _{\nu }S-(\mu +\eta _{\nu })V, \\ \dfrac{\partial R}{\partial t} = \alpha _R\dfrac{\partial ^2 R}{\partial x^2}+\omega I-(\mu +\theta )R. \end{array}\right\} \end{aligned}$$

Moreover, the susceptible individuals get infected after an interaction at the rate $\beta$ and $\beta \psi _{\nu }$ with infected and exposed individuals respectively. The fractions of infected individuals among total population are $\frac{IS}{N}$ and $\frac{ES}{N}$ respectively and therefore, force of infection is,

$$\begin{aligned} \lambda (x, t) = \beta \frac{(I(x, t)+\psi E(x, t))S(x, t)}{N(x, t)}. \end{aligned}$$

The detailed description of the model (1) embedded parameters along with their values are presented in Table 1. The constants $\alpha _{S}, \alpha _{E}, \alpha _{I}, \alpha _{V}$ and $\alpha _{R}$ represents the diffusion rate of respective populations. Moreover, the following no flux boundary conditions are taken into account for the above model:

2 $$\begin{aligned} \left. \begin{array}{ll} \dfrac{\partial S(-2, t)}{\partial x}=\dfrac{\partial E(-2, t)}{\partial x}=\dfrac{\partial I(-2, t)}{\partial x}=\dfrac{\partial V(-2, t)}{\partial x}=\dfrac{\partial R(-2, t)}{\partial x} &{}= 0,\\ \dfrac{\partial S(2, t)}{\partial x}=\dfrac{\partial E(2, t)}{\partial x}=\dfrac{\partial I(2, t)}{\partial x}=\dfrac{\partial V(2, t)}{\partial x}=\dfrac{\partial R(2, t)}{\partial x} = 0.&{} \end{array} \right\} \end{aligned}$$

### Initial conditions

The following set of initial conditions, provided by (3) and (4) are utilized to simulate the model (1). Based on the conditions listed in^6^, the initial conditions given by (4) are chosen as follows:

3 $$\begin{aligned}&\left. \begin{array}{rcl} S(0, x)&{}=&{}S_0 \ge 0,\\ E(0, x)&{}=&{}E_0 \ge 0,\\ I(0, x)&{}=&{}I_0 \ge 0,\\ V(0, x)&{}=&{}V_0 \ge 0,\\ R(0, x)&{}=&{}R_0 \ge 0, \end{array}\right\} \end{aligned}$$

4 $$\begin{aligned}&\left. \begin{array}{rcl} S(0, x)&{}=&{}S_0 \exp \left( -\left( x/0.7\right) ^2\right) ,\\ E(0, x)&{}=&{}E_0\exp \left( -\left( x/0.8\right) ^2\right) ,\\ I(0, x)&{}=&{}I_0\exp \left( -\left( x/0.8\right) ^2\right) ,\\ V(0, x)&{}=&{}V_0\ge 0,\\ R(0, x)&{}=&{}R_0\ge 0, \end{array}\right\} \end{aligned}$$

where $$S_0 = 220870336, E_0 = 16000, I_0 = 4, V_0 = 0$$ and $$R_0 = 0$$.

**Table 1:** Numerical values and description of the model parameters.

Parameters	Meaning	Values [per/day]	References
$\Theta$	Human recruitment rate	8939	Estimated
$\beta$	Contact rate capable of disease transmission	0.4114	^11^
$\kappa$	Transmissibility rate due to exposed class	0.3131	^11^
$\mu$	Natural death rate	$\frac{1}{67.7\times 365}$	^12^
$\mu _{0}$	Disease-induced death in *I*	0.022	^13^
$\gamma$	Flow rate from *E* to *I*	0.0164	^11^
$\psi _{\nu }$	Rate of vaccination	0.0380	^11^
$\eta _{\nu }$	Vaccine waning rate	0.0057	^11^
$\omega$	Recovery rate	0.1000	^11^
$\theta$	Loss of immunity rate	0.1762	^11^

**Figure 1:**
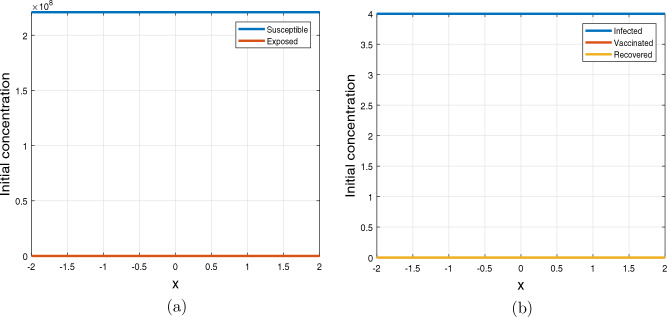
Profile of uniform initial conditions (3) where (**a**) shows the dynamics of susceptible and exposed individuals (**b**) shows the dynamics of infected, vaccinated and recovered population.

**Figure 2:**
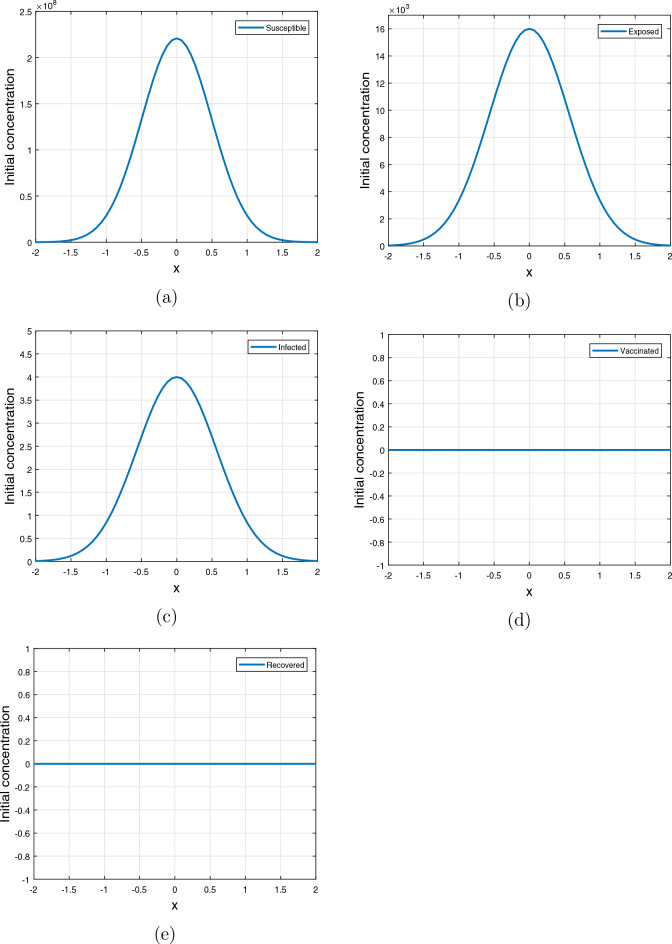
Initial conditions (4) profiles where (**a**) susceptible population, (**b**) exposed population, (**c**) infected population, (**d**) vaccinated population, (**e**) recovered population.

Figures 1 and 2 illustrate profiles of initial conditions. Figure 1 describes initial conditions (3), it has uniform population distribution throughout the domain $[-2, 2]$, for each sub-populations considered in the study (Fig. 4). It is assumed that the proportion of susceptible individuals is greater than all other sub-populations, while the vaccinated and recovered populations are kept at zero. On the other hand, Fig. 4 illustrates initial condition (4) with susceptible, exposed and infected are concentrated at the origin in the center of the domain $\left[ -2, 2\right]$ and decrease exponentially on both sides of the origin while the vaccinated and recovered concentrations are considered zeros in this case.

## Qualitative analysis

The present section deals with qualitative properties of the model (1), including existence and uniqueness, positivity, and boundedness of the solution to assure its global time existence. Moreover, the stability of the model (1) steady states is discussed in detail.

### Well-posedness of model

In the analysis of partial differential equations (PDE), it is important to verify whether the PDE combined with some initial and boundary conditions is well-posed since it claims the solution existence and uniqueness. To establish the well-posedness of a mathematical problem under consideration, the semi-group theory approach is employed. In this context, a Banach space denoted by $X = C(\Omega ; \mathbb {R})$ is considered. This space consists of real-valued continuous functions $\phi$ defined over the set $\Omega$, where $X_{+}$ representing the positive cone of this space. Moreover, the norm $|\phi |_{X}$ is defined as the supremum of $|\phi (x)|$ over all $x\in \Omega$. We assume the existence of a linear operator $A_{\xi }$, defined as follows:

$$\begin{aligned} A_{\xi }(\phi ) = \alpha _{\xi }\Delta \phi , \end{aligned}$$

where $\xi$ stands for the respective model state variables, $\Delta$ is the differential operator $\frac{\partial ^2}{\partial x^2}$ and

$$\begin{aligned} D(A_{\xi }) = \left\{ \phi \in X: \Delta \phi \in X, \frac{\partial \phi }{\partial x} = 0 \ \hbox {on} \ \delta \Omega \right\} . \end{aligned}$$

Based on the well-established knowledge pertaining to the operator $A_{\xi }$, it is known that $A_{\xi }$ serves as the infinitesimal generator of a strongly continuous semigroup denoted by $\{e^{tA_{\xi }}: t\ge 0\}$ consisting of linear operators in the Banach space *X*^14^. Moreover, considering the operator defined as

5 $$\begin{aligned} A(\phi _{1}, \phi _{2}, \phi _{3}, \phi _{4}, \phi _{5}) = \left( \begin{array}{ll} A_{\xi }(\phi _{1})\\ A_{\xi }(\phi _{2})\\ A_{\xi }(\phi _{3})\\ A_{\xi }(\phi _{4})\\ A_{\xi }(\phi _{5}) \end{array}\right) , D(A) = D(A_{\xi })\times D(A_{\xi }). \end{aligned}$$

It can be inferred that the above operator also serves as an infinitesimal generator of a strongly continuous semigroup $\{e^{tA}: t\ge 0\}$ consisting of linear operators in the Banach space $Y = X^{5}$^14^. Here, the Banach space *Y* is coupled with a norm defined by:

$$\begin{aligned} \Vert (\phi _{1}, \phi _{2}, \phi _{3}, \phi _{4}, \phi _{5})\Vert _{Y} = \Vert \phi _{1}\Vert _{Y}+\Vert \phi _{2}\Vert _{Y}+\Vert \phi _{3}\Vert _{Y}+\Vert \phi _{4}\Vert _{Y}+\Vert \phi _{5}\Vert _{Y}, \end{aligned}$$

and $Y_{+} = X_{+}^{5}\subset Y$ indicates a positive cone in the space *Y*. Further, considering *F* as a nonlinear operator given *Y* as:

$$\begin{aligned}\nonumber F(\phi _{1}, \phi _{2}, \phi _{3}, \phi _{4}, \phi _{5}) = \left( \begin{array}{ll} \Theta -\beta (\phi _{3}+\kappa \phi _{2})\dfrac{\phi _{1}}{N}-(\psi _{\nu }+\mu )\phi _{1}+\eta _{\nu }\phi _{4}+\theta \phi _{5}\\ \beta (\phi _{3}+\kappa \phi _{2})\dfrac{\phi _{1}}{N}-(\mu +\gamma )\phi _{2}\\ \gamma \phi _{2} - (\mu +\mu _{0}+\omega )\phi _{3}\\ \psi _{\nu }\phi _{1}-(\mu +\eta _{\nu })\phi _{4}\\ \phi _{3}\omega -(\mu +\theta )\phi _{5} \end{array}\right) , \end{aligned}$$

for $(\phi _{1}, \phi _{2}, \phi _{3}, \phi _{4}, \phi _{5}) \in Y_{+}$. As a result, the model (1) can be rewrite in a comprehensive form as follows:

6 $$\begin{aligned} \dfrac{du}{dt} = Au+F(u), \end{aligned}$$

where

$$\begin{aligned} u(t) = \left( \begin{array}{ll} S(\cdot , t)\\ E(\cdot , t)\\ I(\cdot , t)\\ V(\cdot , t)\\ R(\cdot , t) \end{array}\right) \ \hbox {and} \ u(0) = \left( \begin{array}{ll} S_0(\cdot )\\ E_0(\cdot )\\ I_0(\cdot )\\ V_0(\cdot )\\ R_0(\cdot ) \end{array}\right) \end{aligned}$$

It is obvious that the operator *F* is Lipschitz continuous on $Y_{+}$ due the preserving following property

$$\begin{aligned} \Vert F(x_1)-F(x_2)\Vert \le \mathcal {L}\Vert x_1-x_2\Vert \ \hbox {for all} \ x_1, x_2 \in Y_{+}, \end{aligned}$$

where, $\mathcal {L} = \mu +\mu _0+2(\gamma +\psi _{\nu }+\eta _{\nu }+\theta +\omega )$. Therefore, with the help of Theorem 3.3.3 in^15^, we state the following preposition.

#### Proposition 3.1

*Let* *A* *be an operator defined by* (5), *and* $u_{0} = (S_0, E_0, I_0, V_0, R_0)^T \in Y_{+}$ *be the respective initial condition. It is assumed that there exists a unique continuously differentiable solution* *u*(*t*) *of* (6) *defined on some maximum interval of existence* $[0, \tau ]$, *satisfying the following equation*:

$$\begin{aligned} u(t) = u_0e^{tA}+\int _{0}^{t}e^{(t-\zeta )A}F(u(\zeta ))d\zeta . \end{aligned}$$

### Positivity and boundedness

The subsequent lemma, which has been widely employed in demonstrating the non-negativity of the solution to system (1)^16–18^.

#### Lemma 3.1

*Assume that* $u \in C({\bar{\Omega }}\times [0, \tau ))\bigcap C^{2, 1}(\Omega \times (0, \tau ))$ *fulfil the following conditions*

7 $$\begin{aligned} \left. \begin{array}{ll} \dfrac{\partial }{\partial t}u(x, t)-D\Delta u(x, t) \ge k(x, t)u(x, t), & x\in \Omega , t\in [0, \tau ) \\ \dfrac{\partial }{\partial \textbf{n}}u(x, t) \ge 0, & x\in \delta \Omega , t\in [0, \tau )\\ u(x, 0)\ge 0, & x\in {\bar{\Omega }}, t\in [0, \tau ) \end{array}\right\} \end{aligned}$$

*where in* (7), *the expression* $k(x, t) \in C({\bar{\Omega }}\times [0, \tau ))$ *is a continuous function*. *Then* $u(x, t) \ge 0$ *on* ${\bar{\Omega }}\times [0, \tau )$. *Further*, $u(x, t)>0$ *or* $u(x, t)\equiv 0$ *in* $\Omega \times [0, \tau )$.

Given Lemma 3.1, we can deduce the following result.

#### Proposition 3.2

*The solution* $(S(\cdot ,t), E(\cdot ,t), I(\cdot ,t), V(\cdot ,t), R(\cdot ,t))$ *of model* (1) *is non-negative on* $\Omega \times [0, \tau )$ *under the assumption that the initial condition* $(S(\cdot ), E(\cdot ), I(\cdot ), V(\cdot ), R(\cdot ))$ *is non-negative*.

#### *Proof*

Let $k(x, t) = -\beta (I(x, t)+\kappa E(x, t))\frac{1}{N}-(\mu +\psi _{\nu })$. Then, from the first equation of model (1), we have:

8 $$\begin{aligned} \left. \begin{array}{*{20}l} { \dfrac{\partial }{\partial t}S(x, t)-\alpha _{S}\dfrac{\partial ^2}{\partial x^2}S(x, t)> k(x, t)S(x, t),} & \quad {x \in \Omega , t\in [0, \tau )}\\ \dfrac{\partial }{\partial \textbf{n}}S(x, t) = 0, &\quad x\in \delta \Omega , t\in [0, \tau )\\ S(x, 0) > 0, &\quad x\in \Omega , t\in [0, \tau ) \end{array}\right\} . \end{aligned}$$

It is evident that (8) satisfies the criteria presented in Lemma 3.1. Thus, by applying Lemma 3.1 directly, we obtain $S(x, t)\ge 0$ on $\Omega \times [0, \tau )$. Similar arguments can be applied to prove the non-negativity of the remaining state variables *E*(*x*, *t*), *I*(*x*, *t*), *V*(*x*, *t*), and *R*(*x*, *t*). $\square$

Next, we demonstrate the boundedness of the solution to model (1) to provide the existence of a global solution. This is accomplished through the subsequent result.

#### Theorem 3.2

*The solution* $(S(\cdot ,t), E(\cdot ,t), I(\cdot ,t), V(\cdot ,t), R(\cdot ,t))$ *of the system* (1) *is bounded for all* $t\ge 0.$

#### *Proof*

By adding all equations of model (1), we have

$$\begin{aligned}&\frac{\partial S(x, t)}{\partial t}+\frac{\partial E(x, t)}{\partial t}+\frac{\partial I(x, t)}{\partial t}+\frac{\partial V(x, t)}{\partial t}+\frac{\partial R(x, t)}{\partial t} \\&\quad =\alpha _{S} \frac{\partial ^2 S(x, t)}{\partial x^2}+\alpha _{E}\frac{\partial ^2 E(x, t)}{\partial x^2}+\alpha _{I}\frac{\partial ^2 I(x, t)}{\partial x^2}+\alpha _{V}\frac{\partial ^2 V(x, t)}{\partial x^2}+\alpha _{R}\frac{\partial ^2 R(x, t)}{\partial x^2}\\&\qquad +\Theta -\mu (S(x, t)+E(x, t)+I(x, t)+V(x, t)+R(x, t))-\mu _0I(x, t). \end{aligned}$$

Integrate over $\Omega = [-2,2]$ yields to,

$$\begin{aligned}&\int _{\Omega }\bigg \{\frac{\partial S(x, t)}{\partial t}+\frac{\partial E(x, t)}{\partial t}+\frac{\partial I(x, t)}{\partial t}+\frac{\partial V(x, t)}{\partial t}+\frac{\partial R(x, t)}{\partial t}\bigg \}dx \\&\quad = \int _{\Omega }\bigg \{\alpha _{S} \frac{\partial ^2 S(x, t)}{\partial x^2}+\alpha _{E}\frac{\partial ^2 E(x, t)}{\partial x^2}+\alpha _{I}\frac{\partial ^2 I(x, t)}{\partial x^2}+\alpha _{V}\frac{\partial ^2 V(x, t)}{\partial x^2}+\alpha _{R}\frac{\partial ^2 R(x, t)}{\partial x^2}\bigg \}dx\\&\qquad + \int _{\Omega }\bigg \{\Theta -\mu (S(x, t)+E(x, t)+I(x, t)+V(x, t)+R(x, t))-\mu _0I(x, t)\bigg \}dx. \end{aligned}$$

using conditions (2) we have

$$\begin{aligned} \int _{\Omega }\bigg \{\alpha _{S} \frac{\partial ^2 S(x, t)}{\partial x^2}+\alpha _{E}\frac{\partial ^2 E(x, t)}{\partial x^2}+\alpha _{I}\frac{\partial ^2 I(x, t)}{\partial x^2}+\alpha _{V}\frac{\partial ^2 V(x, t)}{\partial x^2}+\alpha _{R}\frac{\partial ^2 R(x, t)}{\partial x^2}\bigg \}dx = 0. \end{aligned}$$

Therefore,

$$\begin{aligned}&\int _{\Omega }\bigg \{\frac{\partial S(x, t)}{\partial t}+\frac{\partial E(x, t)}{\partial t}+\frac{\partial I(x, t)}{\partial t}+\frac{\partial V(x, t)}{\partial t}+\frac{\partial R(x, t)}{\partial t}\bigg \}dx \\&\quad =\Theta |\Omega |-\mu \int _{\Omega }\bigg \{S(x, t)+E(x, t)+I(x, t)+V(x, t)+R(x, t)\bigg \}dx\\&\qquad -\mu _0\int _{\Omega }I(x, t)dx\\&\quad \le \Theta |\Omega |-\mu \int _{\Omega }\bigg \{S(x, t)+E(x, t)+I(x, t)+V(x, t)+R(x, t)\bigg \}dx\\&\quad =\Theta |\Omega |-\mu \mathcal {N}(t),\\ \dfrac{d}{dt}\mathcal {N}(t)&\quad \le \Theta |\Omega |-\mu \mathcal {N}(t). \end{aligned}$$

It follows that

$$\begin{aligned} 0\ge \mathcal {N}(t)\le \frac{\Theta |\Omega |}{\mu }+\exp (-\mu t)\mathcal {N}(0). \end{aligned}$$

Thus,

$$\begin{aligned} \lim \limits _{t\rightarrow +\infty }\mathcal {N}(t) \le \frac{\Theta |\Omega |}{\mu }. \end{aligned}$$

$$\square$$

### Invariant region

The biological feasible region for the dynamics of model under consideration is

$$\begin{aligned} \Delta \subset \mathbf {{R}}^{5}_{+}, \end{aligned}$$

where,

$$\begin{aligned} \Delta = \bigg \{(S(x, t), E(x, t), I(x, t), V(x, t), R(x, t))\in {\textbf{R}}^{5}_{+}: \mathcal {N}(t) \le \frac{\Theta |\Omega |}{\mu }\bigg \}. \end{aligned}$$

### Steady-states of the model

The COVID-19 model (1) possesses two steady-states. The disease-free equilibrium point is $\xi _{0} = \left( S_{0}, E_{0}, I_{0}, V_{0},R_{0}\right) = \left( \frac{\Theta (\mu +\eta _{\nu })}{\mu (\mu +\eta _{\nu }+\psi _{\nu })}, 0, 0, \frac{\Theta \psi _{\nu }}{\mu (\mu +\eta _{\nu }+\psi _{\nu })}, 0 \right)$ and endemic equilibrium point $\xi _{EE} = \left( S_{*}, E_{*}, I_{*}, V_{*},R_{*}\right)$

$$\begin{aligned} = {\left\{ \begin{array}{ll} S_* = \frac{\Theta (\mu +\eta _{\nu })M_{2}}{(\mu +\eta _{\nu }+\psi _{\nu })[(\mathcal {R}_{0}-1)M_{1}+\mu M_{2}]},\\ \\ E_* = \frac{\Theta (\mu +\theta )(\mu +\mu _{0}+\omega )(\mathcal {R}_{0}-1)}{(\mathcal {R}_{0}-1)M_{1}+\mu M_{2}},\\ \\ I_* = \frac{\gamma \Theta (\mu +\theta )(\mathcal {R}_{0}-1)}{(\mathcal {R}_{0}-1)M_{1}+\mu M_{2}},\\ \\ V_* = \frac{\Theta \psi _{\nu }M_{2}}{(\mu +\eta _{\nu }+\psi _{\nu })[(\mathcal {R}_{0}-1)M_{1}+\mu M_{2}]},\\ \\ R_* = \frac{\gamma \omega \Theta (\mathcal {R}_{0}-1)}{(\mathcal {R}_{0}-1)M_{1}+\mu M_{2}}, \end{array}\right. } \end{aligned}$$

where $M_{1} = (\mu +\omega +\theta )\mu \gamma +(\mu +\theta )(\mu +\omega )\mu +\mu _{0}(\mu +\theta )(\mu +\gamma )$ and $M_{2} = (\mu +\theta )(\mu +\mu _{0}+\omega )+\gamma (\mu +\omega +\theta )$, and $\mathcal {R}_{0}$ is the the basic reproduction number presented in^11^ and given as fallow:

$$\begin{aligned} \mathcal {R}_{0}&=\frac{\beta (\mu +\eta _{\nu })(\gamma +\kappa (\mu +\mu _{0}+\omega ) )}{(\mu +\eta _{\nu }+\psi _{\nu })(\mu +\gamma )(\mu +\mu _{0}+\omega )},\\ \mathcal {R}_{0}&= \mathcal {R}_{1}+\mathcal {R}_{2}, \end{aligned}$$

where,

$$\begin{aligned} \mathcal {R}_{1} = \dfrac{\beta \gamma (\mu +\eta _{\nu }) }{(\mu +\eta _{\nu }+\psi _{\nu })(\mu +\gamma )(\mu +\mu _{0}+\omega )}, \ \hbox {and} \ \mathcal {R}_{2} = \dfrac{\beta \kappa (\mu +\eta _{\nu })}{(\mu +\gamma )(\mu +\eta _{\nu }+\psi _{\nu })}. \end{aligned}$$

### Local stability

#### Theorem 3.3

*If* $\mathcal {R}_0<1$, *the disease free equilibrium* $\xi _0$ *is locally asymptotically stable*.

#### *Proof*

The Jacobian of the system (1) at the DFE point $\xi _0 = (\frac{\Delta }{d}, 0, 0, 0, 0, 0)$ is,

$$\begin{aligned}\nonumber J(\xi _0) =\left( \begin{array}{ccccc} -(\mu +\psi _{\nu } ) &{} -\frac{\beta \kappa (\mu +\eta _{\nu })}{\mu +\eta _{\nu }+\psi _{\nu }} &{} -\frac{\beta (\mu +\eta _{\nu })}{\mu +\eta _{\nu }+\psi _{\nu }} &{} \eta _{\nu } &{} \theta \\ 0 &{} -\gamma -\mu +\frac{\beta \kappa (\mu +\eta _{\nu })}{\mu +\eta _{\nu }+\psi _{\nu }} &{} \frac{\beta (\mu +\eta _{\nu })}{\mu +\eta _{\nu }+\psi _{\nu }} &{} 0 &{} 0 \\ 0 &{} \gamma &{} -\mu -\mu _0-\omega &{}0 &{}0\\ \psi _{\nu } &{} 0 &{} 0 -\mu -\eta _{\nu } &{}0 &{}0\\ 0 &{} 0 &{} \omega &{}0 &{}-\theta -\mu \end{array} \right) \end{aligned}$$

and the associated characteristic polynomial is,

9 $$\begin{aligned} P(\lambda ) = (-\lambda -\theta -\mu )(\lambda +\mu )(\lambda +\mu +\lambda _{\nu }+\psi _{\nu })(a_2\lambda ^2+a_1\lambda +a_0), \end{aligned}$$

where

$$\begin{aligned} a_2&= 1,\\ a_1&= \mu +\mu _0+\omega +(\gamma +\mu )(1-\mathcal {R}_2),\\ a_0&= (\mu +\mu _0+\omega )(\gamma +\mu )(\mu +\eta _{\nu }+\psi _{\nu })(1-\mathcal {R}_0). \end{aligned}$$

The coefficients of the characteristics equation (9) $a_0, a_1, a_2$ are positive, claimed by $\mathcal {R}_0<1$. It fulfill the necessary condition of Routh–Hurwitz stability criteria^19^. Thus the *DFE* point is locally asymptotically stable in $\Omega$ whenever $R_{0}<1$. $\square$

### Linearized stability of endemic equilibrium

To discuss linearized stability of endemic equilibrium i.e. $\xi _{EE}=(S_{*}, E_{*}, I_{*}, V_{*},R_{*})$, the spatio-temporal model (1) will be linearized about $\xi _{EE}$. Let ${\bar{S}}(x, t), {\bar{E}}(x, t), {\bar{I}}(x, t),$ ${\bar{V}}(x, t), {\bar{R}}(x, t)$ are small perturbations such that,

10 $$\begin{aligned} \left. \begin{array}{ll} S(x, t) &{}= {\bar{S}}(x, t)+S_{*}, \\ \\ E(x, t) &{}= {\bar{E}}(x, t)+E_{*}, \\ \\ I(x, t) &{}= {\bar{I}}(x, t)+I_{*}, \\ \\ V(x, t) &{}= {\bar{V}}(x, t)+V_{*}, \\ \\ R(x, t) &{}= {\bar{R}}(x, t)+R_{*}. \end{array} \right\} \end{aligned}$$

Thus, in linearized form spatio-temporal model (1) is presented as:

11 $$\begin{aligned} \left. \begin{array}{ll} \dfrac{\partial S}{\partial t} &{}= \alpha _{S}\dfrac{\partial ^2 S}{\partial x^2}+a_{11}{\bar{S}}+a_{12}{\bar{E}}+a_{13}{\bar{I}}+a_{14}{\bar{V}}+a_{15}{\bar{R}}, \\ \\ \dfrac{\partial E}{\partial t} &{}= \alpha _{E}\dfrac{\partial ^2 S}{\partial x^2}+a_{21}{\bar{S}}+a_{22}{\bar{E}}+a_{23}{\bar{I}}+a_{24}{\bar{V}}+a_{25}{\bar{R}}, \\ \\ \dfrac{\partial I}{\partial t} &{}= \alpha _{I}\dfrac{\partial ^2 I}{\partial x^2}+a_{31}{\bar{S}}+a_{32}{\bar{E}}+a_{33}{\bar{I}}+a_{34}{\bar{V}}+a_{35}{\bar{R}}, \\ \\ \dfrac{\partial V}{\partial t} &{}= \alpha _{V}\dfrac{\partial ^2 V}{\partial x^2}+\psi _{\nu }{\bar{S}}+a_{42}{\bar{E}}+a_{43}{\bar{I}}+a_{44}{\bar{V}}+a_{45}{\bar{R}}, \\ \\ \dfrac{\partial R}{\partial t} &{}= \alpha _{R}\dfrac{\partial ^2 R}{\partial x^2}+a_{51}{\bar{S}}+a_{52}{\bar{E}}+a_{53}{\bar{I}}+a_{54}{\bar{V}}+a_{55}{\bar{R}}, \end{array} \right\} \end{aligned}$$

where,

$$\begin{aligned}\nonumber \begin{array}{ll} &{}a_{11} = -\dfrac{\beta }{N}\left( I_{*}+\kappa E_{*}\right) -\mu -\psi _{\nu }, \ a_{12} = -\dfrac{\kappa \beta }{N}S_{*}, \ a_{13} = -\dfrac{\beta }{N}S_{*}, \ a_{14} = \eta _{\nu }, \\ &{}a_{15} = \theta ,\hspace{0.3cm} a_{21} = \dfrac{\beta }{N}\left( I_{*}+\kappa E_{*}\right) , \hspace{0.3cm} a_{22} = \dfrac{\kappa \beta }{N}S_{*}-\mu -\gamma , \ a_{23} = \dfrac{\beta }{N}S_{*}, \\ &{} a_{24} = 0, \hspace{0.3cm} a_{25} = 0, \hspace{0.3cm} a_{31} = 0, \hspace{0.3cm} a_{32} = \gamma , \hspace{0.3cm} a_{33} = -(\mu +\mu _{0}+\omega ), \hspace{0.3cm} a_{34} = 0, \\ &{}a_{35} = 0, \hspace{0.3cm} a_{41} = \psi _{\nu }, \hspace{0.3cm} a_{42} = 0, \hspace{0.3cm} a_{43} = 0, \hspace{0.3cm} a_{44} = -(\mu +\eta _{\nu }), \hspace{0.3cm} a_{45} = 0,\\ &{}a_{51} = 0, \hspace{0.3cm} a_{52} = 0, \hspace{0.3cm} a_{53}=\omega , \hspace{0.3cm} a_{54}=0, \hspace{0.3cm} a_{55} = -(\mu +\theta ). \end{array} \end{aligned}$$

It is assumed that the linearized model (11) possess Fourier series solution then,

12 $$\begin{aligned} \left. \begin{array}{ll} {\bar{S}}(x, t) &{}= \sum \limits _{k}\beta _{1k}e^{\eta t}\cos (kx), \\ {\bar{E}}(x, t) &{}= \sum \limits _{k}\beta _{2k}e^{\eta t}\cos (kx), \\ {\bar{I}}(x, t) &{}= \sum \limits _{k}\beta _{3k}e^{\eta t}\cos (kx), \\ {\bar{V}}(x, t) &{}= \sum \limits _{k}\beta _{4k}e^{\eta t}\cos (kx), \\ {\bar{R}}(x, t) &{}= \sum \limits _{k}\beta _{6k}e^{\eta t}\cos (kx), \end{array} \right\} \end{aligned}$$

where $k = \dfrac{n\pi }{2}$ and $n\in Z^{+}$, represent wave number for node *n*. Substituting (12) in (11) we have,

13 $$\begin{aligned} \left. \begin{array}{ll} \sum \limits _{k}(a_{11}-\alpha _{S}k^2-\eta )\beta _{1k}+\sum \limits _{k}a_{12}\beta _{2k}+\sum \limits _{k}a_{13}\beta _{3k}+\sum \limits _{k}a_{14}\beta _{4k}+\sum \limits _{k}a_{15}\beta _{5k} &{} = 0, \\ \sum \limits _{k}a_{21}\beta _{1k}+\sum \limits _{k}(a_{22}-\alpha _{E}k^2-\eta )\beta _{2k}+\sum \limits _{k}a_{23}\beta _{3k}+\sum \limits _{k}a_{24}\beta _{4k}+\sum \limits _{k}a_{25}\beta _{5k} &{} = 0, \\ \sum \limits _{k}a_{31}\beta _{1k}+\sum \limits _{k}a_{32}\beta _{2k}+\sum \limits _{k}(a_{33}-\alpha _{I}k^2-\eta )\beta _{3k}+\sum \limits _{k}a_{34}\beta _{4k}+\sum \limits _{k}a_{35}\beta _{5k} &{} = 0, \\ \sum \limits _{k}a_{41}\beta _{1k}+\sum \limits _{k}a_{42}\beta _{2k}+\sum \limits _{k}a_{43}\beta _{3k}+\sum \limits _{k}(a_{44}-\alpha _{V}k^2-\eta )\beta _{4k}+\sum \limits _{k}a_{45}\beta _{5k} &{} = 0, \\ \sum \limits _{k}a_{51}\beta _{1k}+\sum \limits _{k}a_{52}\beta _{2k}+\sum \limits _{k}a_{53}\beta _{3k}+\sum \limits _{k}a_{54}\beta _{4k}+\sum \limits _{k}(a_{55}-\alpha _{R}k^2-\eta )\beta _{5k} &{} = 0. \end{array} \right\} \end{aligned}$$

Therefore, the variational matrix of (6) is,

14 $$\begin{aligned} V = \left( \begin{array}{ccccc} -c_{11} &{} a_{12} &{} a_{13}&{} a_{14}&{} a_{15} \\ a_{21} &{} c_{22}&{} a_{23} &{} a_{24}&{} 0 \\ 0 &{}a_{23}&{}-c_{33}&{} 0&{} 0\\ a_{41} &{}0&{}0&{}-c_{44}&{} 0\\ 0 &{}0&{}0&{} a_{54}&{}-c_{55}\\ \end{array}\right) , \end{aligned}$$

where

$$\begin{aligned} c_{11}&= a_{11}+\alpha _{S}k^2, \\ c_{22}&= a_{22}-\alpha _{E}k^2, \\ c_{33}&= a_{33}+\alpha _{I}k^2, \\ c_{44}&= a_{44}+\alpha _{V}k^2, \\ c_{55}&= a_{55}+\alpha _{R}k^2, \end{aligned}$$

and the associated characteristics polynomial is,

15 $$\begin{aligned} \mathcal {P}(\eta ) = \eta ^5+p_4\eta ^4+p_3\eta ^3+p_2\eta ^2+p_1\eta +p_0, \end{aligned}$$

where

$$\begin{aligned} p_{4}&= k^{2}(\alpha _{S}+\alpha _{E}+\alpha _{I}+\alpha _{V}+\alpha _{R})+4\mu +\mu _{0}+\gamma +\omega +\theta +\eta _{\nu }+\psi _{\nu }+(\mu +\gamma )\\&\quad \times \left( 1-\frac{\mathcal {R}_2}{\mathcal {R}_0}\right) ,\\ p_{3}&=c_{33}c_{44}+c_{33}c_{55}+c_{44}c_{55}+k^{4}\alpha _{S}\alpha _{E}+(k^{2}(\alpha _{I}+\alpha _{V}+\alpha _{R})+3\mu +\mu _{0}+\omega +\eta _{\nu }+\theta )\\&\quad \times \left( \alpha _{E}k^{2}+(\mu +\gamma )\left( 1-\frac{\mathcal {R}_2}{\mathcal {R}_0}\right) \right) +(\mu +\gamma )(\alpha _{S}k^{2}+\mu +\psi _{\nu })\left( 1-\frac{\mathcal {R}_2}{\mathcal {R}_0}\right) +(\mu +\psi _{\nu })\\&\quad \times (k^{2}(\alpha _{E}+\alpha _{I}+\alpha _{V}+\alpha _{R})+2\mu +\mu _{0}+\omega +\theta )+a_{21}(\alpha _{E}k^{2}+\mu +\gamma )+\mu (\eta _{\nu }+\psi _{\nu })\\&\quad +(\mu +\mu _{0}+\omega )\left( \mu \left( 1-\frac{\mathcal {R}_1}{\mathcal {R}_0}\right) -\gamma \left( \frac{\mathcal {R}_1}{\mathcal {R}_0}\right) \right) ,\\ p_{2}&= \left( 2a_{21}(k^{2}\alpha _{E}+\mu +\gamma )+(k^{2}\alpha _{S}+\mu +\psi _{\nu })\left( \alpha _{E}k^{2}+(\mu +\gamma )\left( 1-\frac{\mathcal {R}_2}{\mathcal {R}_0}\right) \right) \right) \\&\quad \times (k^{2}(\alpha _{I}+\alpha _{V}+\alpha _{R})+3\mu +\mu _{0}+\omega +\eta _{\nu }+\theta )+(k^{2}\alpha _{R}+\mu +\theta )(k^{2}\alpha _{V}+\mu +\eta _{\nu })\\&\quad \times \left( \alpha _{E}k^{2}+(\mu +\gamma )\left( 1-\frac{\mathcal {R}_2}{\mathcal {R}_0}\right) \right) +k^{4}(k^{2}\alpha _{S}+\mu +\psi _{\nu })(\alpha _{V}\alpha _{R}+\alpha _{I}(\alpha _{V}+\alpha _{R}))\\&\quad +k^{2}(k^{2}\alpha _{S}+\mu +\psi _{\nu })(\alpha _{I}(2\mu +\theta )+\alpha _{R}(2\mu +\omega )+\alpha _{V}(2\mu +\theta +\omega ))-c_{33}c_{44}c_{55}\\&\quad +\eta _{\nu }(k^{2}(\alpha _{I}+\alpha _{R})+\theta +2\mu +\mu _{0}+\omega )+(k^{2}\alpha _{V}+\mu +\eta _{\nu })(k^{2}\alpha _{I}+\mu +\mu _{0}+\omega )\\&\quad \times \left( \alpha _{E}k^{2}+(\mu +\gamma )\left( 1-\frac{\mathcal {R}_2}{\mathcal {R}_0}\right) \right) +\mu _{0}(k^{2}\alpha _{S}+\mu +\psi _{\nu })(k^{2}(\alpha _{R}+\alpha _{V})+2\mu +\theta )\\&\quad +(k^{2}\alpha _{R}+\mu +\theta )(k^{2}\alpha _{I}+\mu +\mu _{0}+\omega ))\left( \alpha _{E}k^{2}+(\mu +\gamma )\left( 1-\frac{\mathcal {R}_2}{\mathcal {R}_0}\right) \right) +\eta _{\nu }\psi _{\nu }\\&\quad \times \left( \frac{\beta \kappa (\mu +\eta _{\nu })}{\mathcal {R}_{0}(\mu +\eta _{\nu }+\psi _{\nu })}\right) +(k^{2}\alpha _{S}+\mu +\psi _{\nu })(\theta (2\mu +\omega )+\mu (3\mu +2\omega ))\\&\quad -\left( \eta _{\nu }\psi _{\nu }+\frac{\beta \kappa (\mu +\eta _{\nu })}{\mathcal {R}_{0}(\mu +\eta _{\nu }+\psi _{\nu })}\right) (k^{2}(\alpha _{I}+\alpha _{V}+\alpha _{R})+3\mu +\mu _{0}+\omega +\eta _{\nu }+\theta ),\\ \end{aligned}$$

$$\begin{aligned} p_{1}&= \alpha _{E}k^{2}(\alpha _{I}k^{2}+\mu +\mu _{0}+\omega )(\alpha _{V}k^{2}+\mu +\eta _{\nu })(\alpha _{R}k^{2}+\mu +\theta )+k^{6}\alpha _{I}\alpha _{V}\alpha _{R}\left( 1-\frac{\mathcal {R}_2}{\mathcal {R}_0}\right) \\&\quad +k^{4}(\mu +\gamma )(\alpha _{I}\alpha _{V}(\mu +\theta )+\alpha _{I}\alpha _{R}(\mu +\eta _{\nu })+\alpha _{R}\alpha _{V}(\mu 
+\mu _{0}+\omega ))\left( 1-\frac{\mathcal {R}_2}{\mathcal {R}_0}\right) +k^{2}(\mu +\gamma )\\&\quad \times (\alpha _{I}(\mu +\theta )(\mu +\eta _{\nu })+\alpha _{V}(\mu +\theta )(\mu +\mu _{0}+\omega )+\alpha _{R}(\mu +\eta _{\nu })(\mu +\mu _{0}+\omega ))\left( 1-\frac{\mathcal {R}_2}{\mathcal {R}_0}\right) \\&\quad -\frac{k^{2}\alpha _{V}\beta \kappa \mu \omega \eta _{\nu }}{\mathcal {R}_{0}(\mu +\eta _{\nu }+\psi _{\nu })}+k^{2}\alpha _{R}(\mu +\gamma )(\mu +\eta _{\nu })(\mu +\mu _{0}+\omega ))\left( 1-\frac{\mathcal {R}_2}{\mathcal {R}_0}\right) +(\mu +\gamma )(\mu +\theta )\\&\quad \times (\mu +\eta _{\nu })(\mu +\mu _{0}+\omega ))\left( 1-\frac{\mathcal {R}_2}{\mathcal {R}_0}\right) +k^{4}(\alpha _{S}+\mu +\psi _{\nu })(\mu +\gamma )(\alpha _{I}\alpha _{R}+\alpha _{I}\alpha _{V}+\alpha _{R}\alpha _{V})\\&\quad \times \left( 1-\frac{\mathcal {R}_2}{\mathcal {R}_0}\right) +k^{2}(\alpha _{I}(2\mu +\theta +\eta _{\nu })+\alpha _{V}(2\mu +\omega +\mu _{0}+\omega )+\alpha _{R}(2\mu +\omega +\eta _{\nu }+\mu _{0}))\\&\quad \times (\alpha _{S}k^{2}+\mu +\psi _{\nu })(\mu +\gamma )\left( 1-\frac{\mathcal {R}_2}{\mathcal {R}_0}\right) + (\alpha _{S}k^{2}+\mu +\psi _{\nu })\left( \alpha _{E}k^{2}+(\mu +\gamma )\left( 1-\frac{\mathcal {R}_2}{\mathcal {R}_0}\right) \right) \\&\quad \times ((2\mu +\eta _{\nu }+\theta )(\mu +\mu _{0}+\omega )+(\mu +\theta )(\mu +\eta _{\nu }))+\alpha _{E}k^{4}(\alpha _{S}k^{2}+\mu +\psi _{\nu })\mu _{0}(\alpha _{V}+\alpha _{R})\\&\quad \times \alpha _{E}k^{4}(\alpha _{S}k^{2}+\mu +\psi _{\nu })(k^{2}(\alpha _{V}\alpha _{R}+\alpha _{I}(\alpha _{V}+\alpha _{R})+\eta _{\nu }(\alpha _{I}+\alpha _{R})+(2\mu +\theta )\alpha _{I})+\alpha _{E}k^{4}\\&\quad \times (\alpha _{S}k^{2}+\mu +\psi _{\nu })((2\mu +\omega +\theta )\alpha _{V}+(2\mu +\omega )\alpha _{R}),\\ p_{0}&= (c_{44}c_{55}(k^{2}\alpha _{S}+\mu )+c_{55}(c_{44}+\eta _{\nu })\psi _{\nu })(\mu +\gamma )\alpha _{I}k^{2}\left( 1-\frac{\mathcal {R}_{2}}{\mathcal {R}_0}\right) +a_{21}(\alpha _{V}k^{2}+\mu +\eta _{\nu })\\&\quad \times (\omega (\alpha _{E}\alpha _{R}k^{4}+\mu (\mu +\gamma +\theta )) + (\alpha _{E}k^2+\mu +\gamma )(\alpha _{R}k^2+\mu +\theta )(\alpha _{I}k^2+\mu +\mu _{0}))+\omega \\&\quad \times a_{21}(\alpha _{V}k^{2}+\mu +\eta _{\nu })(\alpha _{R}(\mu +\gamma )+\alpha _{E}(\mu +\theta )). \end{aligned}$$

Thus, the coefficients $P_i$ for $i = 0, 1, 2, 3, 4$ of the characteristics polynomial (15) are positive if and only if $\mathcal {R}_0>1$. Furthermore, the necessary conditions of Routh-Huwritz stability criteria^19^, for degree five polynomial, i.e. $p_4p_3p_2>p_2^2+p_4^2p_1$ and $(p_4p_1-p_0)(p_4p_3p_2-p_2^2-b_4^2p_1)>p_0(p_4p_3-p_2)^2+p_1p_0^2$ holds. Therefore, the endemic equilibrium $\xi _{EE}$ is locally asymptotically stable in $\Omega$ whenever $\mathcal {R}_0>1$.

### Global stability

This section presents the global asymptotical stability of the reaction–diffusion system (1) equilibria. The Lyapunov stability theory is used and constructs a suitable Lyapunov function. This theory was presented by a Russian mathematician and until now used as a strong and fundamental tool for investigating the stability of nonlinear dynamical systems^20^. Moreover, this approach does not requires the exact solution of the system examined for stability. We proceed with the following result.

#### Theorem 3.4

*If* $\mathcal {R}_0<1$, *the disease-free equilibrium* $\xi _{0}$ *of the model* (1) *is stable globally asymptotically*.

#### *Proof*

In order to establish the result, we define a Lyapunov functional as follows:

$$\begin{aligned} \mathcal {V}(t)&= \int _{\Omega }\bigg \{g_1E(x, t)+ \int _{t_0}^{g_1(\mu +\gamma )t}E\left( x, \frac{\sigma }{g_1(\mu +\gamma )}\right) d\sigma +\int _{(\mu +\gamma )t}^{\tau }E\left( x, \frac{\sigma }{\mu +\gamma }\right) d\sigma \bigg \}dx\\&\quad +g_2\int _{\Omega }I(x,t)dx, \end{aligned}$$

where $g_1 = \dfrac{\mu +\eta _{\nu }}{\mu +\eta _{\nu }+\psi _{\nu }}$, $g_2 = \dfrac{\beta g_1}{\mu +\mu _0+\omega }$ and $\tau$ indicates the maximumal time.

The time derivative of $\mathcal {V}(t)$ is

$$\begin{aligned}\nonumber \dfrac{d}{dt}\mathcal {V}(t)&= \int _{\Omega }\bigg \{g_1\alpha _{E}\dfrac{\partial ^2 E(x, t)}{\partial x^2}+g_1\beta (I(x, t)+\kappa E(x, t))\dfrac{S(x, t)}{N(x, t)}\bigg \}dx\\&\quad -g_1(\mu +\gamma )\int _{\Omega }E(x,t) dx+g_1(\mu +\gamma )\int _{\Omega }E\left( x, t\right) dx-(\mu +\gamma )\int _{\Omega }E\left( x, t\right) dx\\&\quad +g_2\int _{\Omega }\biggl \{\alpha _{E}\dfrac{\partial ^2 I(x, t)}{\partial x^2}+\gamma E(x, t)-(\mu +\mu _0+\omega )I(x,t)\biggr \}dx. \end{aligned}$$

Using condition stated in (2) we have

$$\begin{aligned} \int _{\Omega }\bigg \{g_1 \alpha _{E}\dfrac{\partial ^2 E(x, t)}{\partial x^2}+g_2\alpha _{I}\dfrac{\partial ^2 I(x, t)}{\partial x^2}\bigg \}dx = 0. \end{aligned}$$

Since,

$$\begin{aligned} S(x,t)\le N(x, t) \ \hbox {for all} \ t\ge 0, x\in \Omega . \end{aligned}$$

Therefore,

$$\begin{aligned}\nonumber \dfrac{d}{dt}\mathcal {V}(t)&\le \int _{\Omega }\bigg (\bigg \{g_1\beta \kappa +g_2\gamma -(\mu +\gamma )\bigg \}E(x,t) +\bigg \{g_1\beta -g_2(\mu +\mu _0+\omega )\bigg \}I(x,t)\bigg )dx, \nonumber \\&= \int _{\Omega }\bigg \{g_1\beta \kappa +\frac{\beta g_1}{(\mu +\mu _0+\omega )}\gamma -(\mu +\gamma )\bigg \}E(x,t)dx,\\&\le (\mu +\gamma )\int _{\Omega }\bigg \{g_1\left( \frac{\beta (\gamma +\kappa (\mu +\mu _0+\omega ))}{(\mu +\gamma )(\mu +\mu _0+\omega )}\right) -1\bigg \}E(x,t)dx,\\&=(\mu +\gamma )(\mathcal {R}_0-1)\int _{\Omega }E(x,t)dx. \end{aligned}$$

Clearly, $\dfrac{d}{dt}\mathcal {V}(t) \le 0$ for all $t\ge 0$ and $x\in \Omega$ if and only if $\mathcal {R}_0<1$. Moreover $\dfrac{d}{dt}\mathcal {V}(t) = 0$ if and if $E(x, t) \rightarrow 0$, for all $t\ge 0$ and $x\in \Omega$. It follows from system (1) that $I(x, t) \rightarrow 0, R(x, t) \rightarrow 0$, $S(x, t)\rightarrow \frac{\Theta (\mu +\eta _{\nu })}{\mu (\mu +\eta _{\nu }+\psi _{\nu })}$ and $V(x,t) \rightarrow \frac{\Theta \psi _{\nu }}{\mu (\mu +\eta _{\nu }+\psi _{\nu })}$. Thus, $(S, E, I, V, R) \rightarrow \left( \frac{\Theta (\mu +\eta _{\nu })}{\mu (\mu +\eta _{\nu }+\psi _{\nu })}, 0, 0, \frac{\Theta \psi _{\nu }}{\mu (\mu +\eta _{\nu }+\psi _{\nu })}, 0 \right)$. Therefore, the largest compact invariant set in $\bigg \{(S, E, I, V, R): \dfrac{d}{dt}\mathcal {V}(t) = 0 \bigg \}$ is being $\xi _0$. By applying the widely used LaSalle’s invariance principle presented in^21^, concluded the disease-free equilibrium $\xi _0$ of spatio-temporal model (1) is globally asymptotically stable if $\mathcal {R}_0<1$. $\square$

## Numerical methods

The present section deals with the numerical solution of spatio-temporal epidemic vaccine model (1). For this purpose, two numerical schemes are proposed namely the finite difference operator splitting (FDOSM) and mesh-free scheme based on multi-quadratic radial basis function is take into account. In the first method, the finite-difference approximation is used along with the operator-splitting technique and in the second method radial basis function approximation is implemented with operator-splitting technique. The stability and positivity of these numerical methods for an epidemic spatio-temporal model can be found in^4,6,22^. The aim of the study is to analyze the efficiency and effectiveness of the proposed schemes for epidemic models. The Operator-splitting technique is very useful for solving non-linear spatio-temporal partial differential equations since it effectively handles the complexity and non-linearity of reaction–diffusion equations. By implementing this technique, the proposed model (1) can be split into two sub-systems, the nonlinear reaction system of equations given by

16 $$\begin{aligned} \left. \begin{array}{ll} \dfrac{1}{2}\dfrac{\partial S}{\partial t} &{}= \Theta -\beta (I+\kappa E)\dfrac{S}{N}-(\psi _{\nu }+\mu )S+\eta _{\nu }V+\theta R, \\ \dfrac{1}{2}\dfrac{\partial E}{\partial t} &{}= \beta (I+\kappa E)\dfrac{S}{N}-(\mu +\gamma )E, \\ \dfrac{1}{2}\dfrac{\partial I}{\partial t} &{}= \gamma E - (\mu +\mu _{0}+\omega )I, \\ \dfrac{1}{2}\dfrac{\partial V}{\partial t} &{}= \psi _{\nu }S-(\mu +\eta _{\nu })V, \\ \dfrac{1}{2}\dfrac{\partial R}{\partial t} &{}= \omega I-(\mu +\theta )R, \\ \end{array} \right\} \end{aligned}$$

and linear diffusion system of equations,

17 $$\begin{aligned} \left. \begin{array}{ll} \dfrac{1}{2}\dfrac{\partial S}{\partial t} &{}= \alpha _S\dfrac{\partial ^2 S}{\partial x^2}, \\ \dfrac{1}{2}\dfrac{\partial E}{\partial t} &{}= \alpha _E\dfrac{\partial ^2 E}{\partial x^2},\\ \dfrac{1}{2}\dfrac{\partial I}{\partial t} &{}= \alpha _I\dfrac{\partial ^2 I}{\partial x^2},\\ \dfrac{1}{2}\dfrac{\partial V}{\partial t} &{}= \alpha _V\dfrac{\partial ^2 V}{\partial x^2}, \\ \dfrac{1}{2}\dfrac{\partial R}{\partial t} &{}= \alpha _R\dfrac{\partial ^2 R}{\partial x^2}.\\ \end{array} \right\} \end{aligned}$$

### Operator splitting based time descritization

The time descretization of the proposed model (1) can be done in two steps. Initially, using first-order time difference by taking half time step $\frac{1}{2}dt$ from 0 to $\frac{1}{2}dt$. The system (16) can be written in descretized form as follow:

18 $$\begin{aligned} \left. \begin{array}{ll} S^{n+\frac{1}{2}}_{j} &{}= S^{n}_{j}+dt\left( \Theta -\beta (I^{n}_{j}+\kappa E^{n}_ {j})\dfrac{S^{n}_{j}}{N}-(\psi _{\nu }+\mu )S^{n}_{j}+\eta _{\nu }V^{n}_{j}+\theta R^{n}_{j}\right) ,\\ E^{n+\frac{1}{2}}_{j} &{}= E^{n}_{j}+dt\left( \beta (I^{n}_{j}+\kappa E^{n}_{j})\dfrac{S^{n}_{j}}{N}-(\mu +\gamma )E^{n}_{j}\right) ,\\ I^{n+\frac{1}{2}}_{j} &{}= I^{n}_{j}+dt\left( \gamma E^{n}_{j} - (\mu +\mu _{0}+\omega )I^{n}_{j}\right) ,\\ V^{n+\frac{1}{2}}_{j} &{}= V^{n}_{j}+dt\left( \psi _{\nu }S^{n}_{j}-(\mu +\eta _{\nu })V^{n}_{j}\right) ,\\ R^{n+\frac{1}{2}}_{j} &{}= R^{n}_{j}+dt\left( \omega I^{n}_{j}-(\mu +\theta )R^{n}_{j}\right) . \end{array} \right\} \end{aligned}$$

Next, half time step is taken from $\frac{1}{2}dt$ to *dt* and using first-order time difference, the system (17) is represented in descretized form as:

19 $$\begin{aligned} \left. \begin{array}{ll} S^{n+1}_{j} &{}= S^{n+\frac{1}{2}}_{j}+\alpha _{S}dt\dfrac{\partial ^2 S^{n+\frac{1}{2}}_{j}}{\partial x^2},\\ E^{n+1}_{j} &{}= E^{n+\frac{1}{2}}_{j}+\alpha _{E}dt\dfrac{\partial ^2 E^{n+\frac{1}{2}}_{j}}{\partial x^2},\\ I^{n+1}_{j} &{}= I^{n+\frac{1}{2}}_{j}+\alpha _{I}dt\dfrac{\partial ^2 I^{n+\frac{1}{2}}_{j}}{\partial x^2},\\ V^{n+1}_{j} &{}= V^{n+\frac{1}{2}}_{j}+\alpha _{V}dt\dfrac{\partial ^2 V^{n+\frac{1}{2}}_{j}}{\partial x^2},\\ R^{n+1}_{j} &{}= R^{n+\frac{1}{2}}_{j}+\alpha _{R}dt\dfrac{\partial ^2 R^{n+\frac{1}{2}}_{j}}{\partial x^2}. \end{array} \right\} \end{aligned}$$

### Space descretization

The spatial derivatives in (19) will be approximated in two ways, with the conventional second-order finite-difference and with multi-quadratic radial basis function approximation, describes as follow:

#### Space descretization with finite-difference

The finite-difference approximation for second order derivative is defined as:

20 $$\begin{aligned} \dfrac{\partial ^2 \xi ^{n+\frac{1}{2}}_j}{\partial x^2} = \frac{\xi ^{n+\frac{1}{2}}_{j-1}-2\xi ^{n+\frac{1}{2}}_{j}+\xi ^{n+\frac{1}{2}}_{j+1} }{dx^2}, \end{aligned}$$

where $\xi$ indicate the variables *S*, *E*, *I*, *V* and *R*.

#### Space descretization with MQ RBF

In this scenario a set of *K* centers, $x_1, x_2, x_3, \ldots , x_K$ in $\mathbf {{R}^{d}}$ space is considered. Then, the RBF interpolation of a given function $f({{\textbf {x}}})$ is expressed as:

$$\begin{aligned} P({{\textbf {x}}}) = \sum _{j=1}^{K}\alpha _{j}\phi (\Vert {{\textbf {x}}}-x_{j}\Vert _{2}) = \sum _{j=1}^{K}\alpha _{j}\phi (r), \quad {{\textbf {x}}}\in \mathbf {{R}^{d}}. \end{aligned}$$

The function $\phi (r)$ is termed as radial basis function (RBF), defined for $r\ge 0$ where, $r = \sqrt{(x_{i}-x_{j})^2+c^2}\ge 0$, and $i, j = 1, 2, 3, \ldots , K$, while the parameter *c* is known as shape parameter and not necessary to involve RBF interpolation approximation. Moreover, the coefficients $\alpha _{j}$ in the above expansion can be computed from the following interpolation condition,

$$\begin{aligned} P(x_{i}) = f_{i}, \end{aligned}$$

at a set of nodal points $x_{i}$ for $i = 1, 2, 3, \ldots , M$. In our case, the centers and collocation points are considered similar, utilizing the RBF interpolation yields a given partial differential equation to the following system of linear equations,

$$\begin{aligned} {{\textbf {B}}}\varvec{\alpha } = {{\textbf {f}}}, \end{aligned}$$

where $\varvec{\alpha }$ is a $K \times 1$ vector having entries $\alpha _{j}$ for $j = 1, 2, \ldots , K$ and ${{\textbf {B}}}$ is $K \times K$ interpolation matrix with entries, $\phi _{ij} = \phi (\Vert x_{i}-x_{j}\Vert _{2}) = \sqrt{(x_{i}-x_{j})^2+c^2}, \quad i, j = 1, 2, 3, \ldots , K.$ i.e,

$$\begin{aligned}\nonumber {{\textbf {B}}} = \left( \begin{array}{llll} \phi (\Vert x_{1}-x_{1}\Vert _{2}) &{} \phi (\Vert x_{1}-x_{2}\Vert _{2}) &{}\cdots &{}\phi (\Vert x_{1}-x_{K}\Vert _{2})\\ \\ \phi (\Vert x_{2}-x_{1}\Vert _{2}) &{} \phi (\Vert x_{2}-x_{2}\Vert _{2}) &{}\cdots &{}\phi (\Vert x_{2}-x_{K}\Vert _{2})\\ \\ \vdots &{} \vdots &{}\vdots &{}\vdots \\ \\ \\ \phi (\Vert x_{K}-x_{1}\Vert _{2}) &{} \phi (\Vert x_{K}-x_{2}\Vert _{2}) &{}\cdots &{}\phi (\Vert x_{K}-x_{K}\Vert _{2})\\ \\ \end{array} \right) , \end{aligned}$$

and

$$\begin{aligned} {{\textbf {f}}} = \left[ f_1, f_2, \ldots , f_K \right] ^{t}. \end{aligned}$$

Moreover, the derivative of a function $f({\textbf{x}})$ can be approximated using RBF as fallow:

$$\begin{aligned} \mathcal {L}P({\textbf{x}}) = \sum _{j=1}^{K}\alpha _{j}\mathcal {L}\phi (r) = {\textbf{B}}_{\textbf{d}}\varvec{\alpha }, \end{aligned}$$

where

$$\begin{aligned}\nonumber \mathbf {B_d}= & {} \left( \begin{array}{llll} \mathcal {L}\phi (\Vert x_{1}-x_{1}\Vert _{2}) &{} \mathcal {L}\phi (\Vert x_{1}-x_{2}\Vert _{2}) &{}\cdots &{}\mathcal {L}\phi (\Vert x_{1}-x_{K}\Vert _{2})\\ \\ \mathcal {L}\phi (\Vert x_{2}-x_{1}\Vert _{2}) &{} \mathcal {L}\phi (\Vert x_{2}-x_{2}\Vert _{2}) &{}\cdots &{}\mathcal {L}\phi (\Vert x_{2}-x_{K}\Vert _{2})\\ \\ \vdots &{} \vdots &{}\vdots &{}\vdots \\ \\ \\ \mathcal {L}\phi (\Vert x_{K}-x_{1}\Vert _{2}) &{} \mathcal {L}\phi (\Vert x_{K}-x_{2}\Vert _{2}) &{}\cdots &{}\mathcal {L}\phi (\Vert x_{K}-x_{K}\Vert _{2})\\ \\ \end{array} \right) ,\\ \varvec{\alpha }= & {} [\alpha _1, \alpha _2, \ldots , \alpha _K], \nonumber \end{aligned}$$

and $\mathcal {L}$ represent the derivative operator of first or second-order, defined as follow:

$$\begin{aligned} \mathcal {L}(\xi ) = \dfrac{\partial ^2 \xi }{\partial x^2} \quad \hbox {if} \quad \xi \in \Omega ,\\ \mathcal {L}(\xi ) = \dfrac{\partial \xi }{\partial x} \quad \hbox {if} \quad \xi \in \partial \Omega . \end{aligned}$$

The symbols $\Omega$ represent the interior and $\partial \Omega$ denotes the boundary of the domain of study. Now, the proposed procedure based on MQ RBF known as a meshless method, is applied to solve the spatio-temporal vaccine model (1). It has been proven that this method have significant approximation capabilities. The attractive feature of the meshless method is that it is easily extendable to higher dimensions in both cases on scattered and uniform data. By utilizing the RBF approximation for the spatial derivatives in (19), the system of linear equations (19) for the half time step $\frac{1}{2}dt$ to *dt* can be expressed as:

21 $$\begin{aligned} {{\textbf{B}}_\textbf{d}{\varvec{\alpha }}^{\textbf{n}+\frac{\textbf{1}}{\textbf{2}}}_{\textbf{r}}} = {\textbf{F}}, \end{aligned}$$

where

$$\begin{aligned} {{\varvec{\alpha }}^{\textbf{n}+\frac{\textbf{1}}{\textbf{2}}}_{\textbf{r}}} = \left[ \alpha ^{n+\frac{1}{2}}_{r1}, \alpha ^{n+\frac{1}{2}}_{r2}, \ldots , \alpha ^{n+\frac{1}{2}}_{rK} \right] , \ \hbox {for} \ r = 1, 2, 3, 4, 5, \end{aligned}$$

and ${\textbf{F}}$ is a matrix with entries $f_{i}$, defined below:

22 $$\begin{aligned} f_{i} = \xi ^{n+1}_{i} = \xi ^{n+\frac{1}{2}}_{i}+dt\alpha _{\xi }\dfrac{\partial ^2 \xi ^{n+\frac{1}{2}}_{i}}{\partial x^2}, \ i = 1, 2, \ldots , K, \end{aligned}$$

where $\xi$ indicates *S*, *E*, *I*, *V* and *R* and,

23 $$\begin{aligned} \xi ^{n+\frac{1}{2}}_{i}= & {} \sum _{j=1}^{K}\alpha ^{n+\frac{1}{2}}_{rj}\phi (\Vert x_{i}-x_{j}\Vert _2), \ i = 1, 2, \ldots , K, r = 1, 2, 3, 4, 5, \end{aligned}$$

24 $$\begin{aligned} \frac{\partial ^2 \xi ^{n+\frac{1}{2}}_{i}}{\partial x^2}= & {} \sum _{j=1}^{K}\alpha ^{n+\frac{1}{2}}_{rj}\frac{\partial ^2}{\partial x^2}\phi (\Vert x_{i}-x_{j}\Vert _2). \end{aligned}$$

The entries of matrix $\textbf{B}_\textbf{d}$ are

$$\begin{aligned} b_{ij} = \mathcal {L}\phi (\Vert x_{i}-x_{j}\Vert _2), \ i, j = 1, 2, \ldots , K. \end{aligned}$$

The coefficients $\alpha ^{n+\frac{1}{2}}_{rj}$ for $r = 1,2,3, 4,5$ and $j = 1, 2, 3, \ldots , K$ are computed from (21). The system of equations (21) can be solved either by using standard inversion scheme, LU-factorization or by Gauss-elimination technique.

## Simulation and discussion

**Figure 3:**
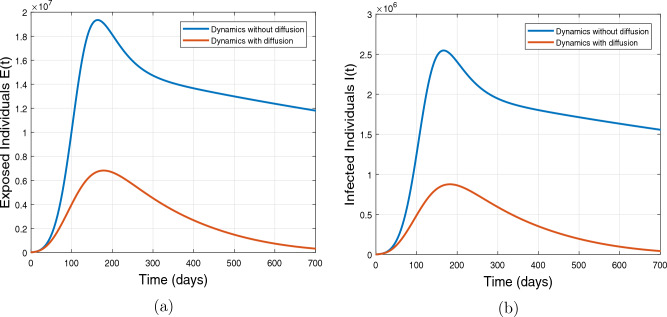
Impact of diffusion on the exposed and infectious individuals (**a**) shows profile of the exposed population and subplot (**b**) depicts the dynamics of infectious individuals with and without diffusion.

The reaction–diffusion COVID-19 vaccine model (1) is simulated by using the proposed iterative schemes FDOSM and RBF as discussed previously. The simulation results are performed in **Matlab** version R2022b for the time period 0–700 days. The coefficients of diffusivity are assumed as $\alpha _S = 0.00005, \alpha _E = 0.0005, \alpha _I = 0.001, \alpha _V = 0.001$ and $\alpha _R = 0$. Initially, the model (1) is simulated for the values of the embedded parameters provided in Table 1 with and without diffusion, in order to analyze the role of diffusion on the dynamic of the COVID-19 epidemic. In the case of without diffusion, the coefficients of diffusivity are considered as $\alpha _{S} = 0, \alpha _{E} = 0, \alpha _{I} = 0, \alpha _{V} = 0, \alpha _{R} = 0$. Figure 3 describes the dynamics of symptomatic and exposed individuals with and without diffusion. A notable observation is that the presence of diffusion leads to a significant reduction in the number of infected individuals compared to the scenario without diffusion. According to biological facts, diffusion phenomena help to restrict the public gathering, as a result, it yields lower chances of getting the infection.

### Simulation using FDOSM

In this subsection, model (1) simulation is performed with and without diffusion by utilizing a finite difference operator-splitting approach. For this purpose, the time step is taken $\Delta t = 0.028$ and corresponds to the number of spatial points ${{\textbf {N}}} = 60$. The spatial step is $\Delta x = 0.06$ which is chosen based on Von Neumann stability criteria presented in^23^. The simulation is carried out for both uniform and nonuniform initial conditions given in Eqs. (3) and (4) respectively.

#### Simulation based on uniform initial condition (3)

**Figure 4:**
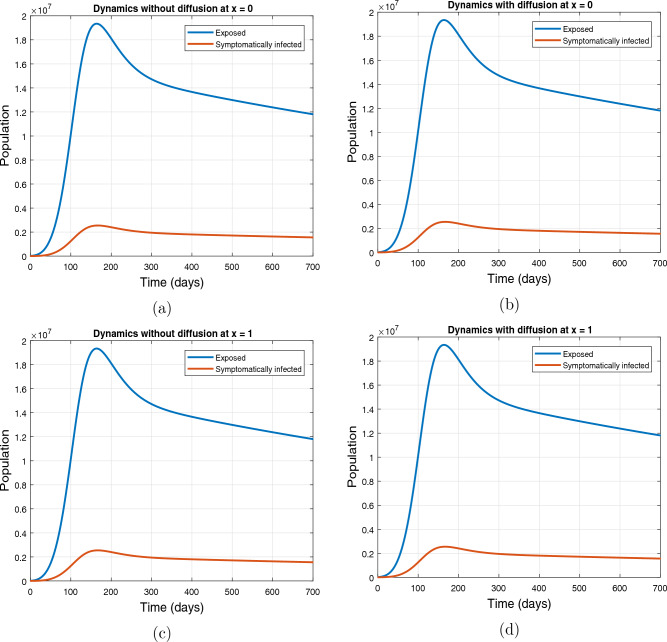
Profiles of exposed and symptomatically infected individuals with and without diffusion where the subplots (**a**,**b**) depict the results at $x=0$, and the subplots (**c**,**d**) show the dynamics at $x=1$.

This part of the paper presents the dynamics of exposed and symptomatically infected populations based on uniform initial conditions (3). The simulation is performed with and without diffusion at $x=0$ and $x=1$. The resulting plot is depicted in Fig. (4) where the subplots (a) and (b) demonstrate the dynamical aspects of exposed and infected classes at $x=0.0$, and the subplots (c) and (d) present the population dynamics at $x=1.0$. The values of parameters used in the simulation are given in Table 1. The population is a spatially uniformly distributed due to the initial condition (3). Therefore, one can observe similar behavior with and without diffusion at $x=0.0$ and as well as at $x=1.0$.

#### Simulation based on non-uniform initial condition at $x=0$

**Figure 5:**
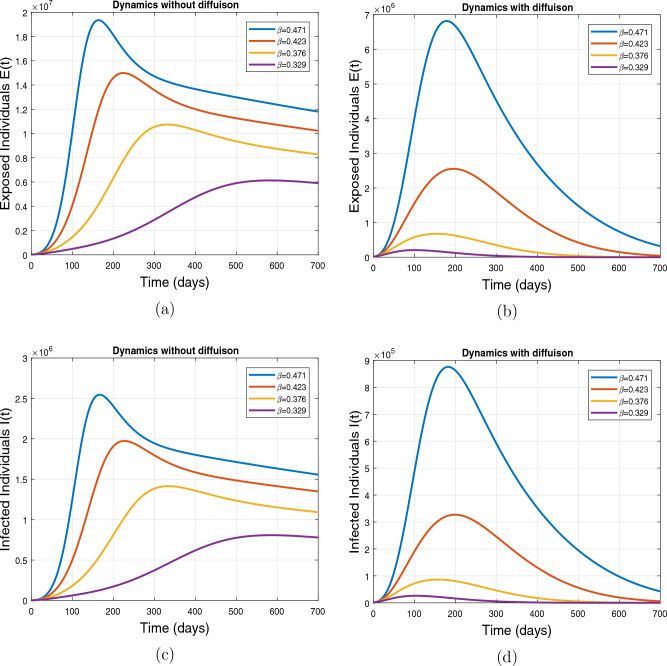
Impact of effective contact rate $\beta$ with and without diffusion at $x=0$. The subplots (**a**,**b**) show the dynamics of exposed individuals and the subplots (**c**,**d**) depict the dynamics of infectious individuals.

**Figure 6:**
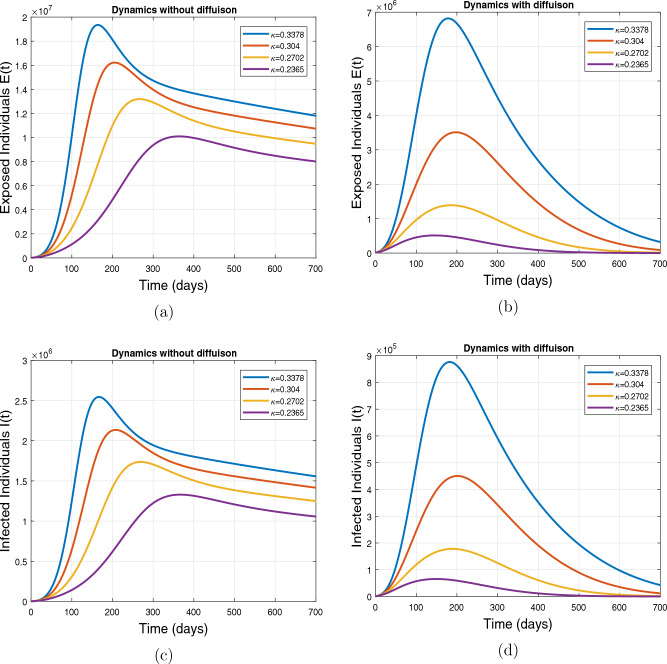
Impact of effective contact rate $\kappa$, with and without diffusion at $x=0.0$ on exposed individuals shown in subplots (**a**,**b**) and on infected individuals shown in subplots (**c**,**d**).

**Figure 7:**
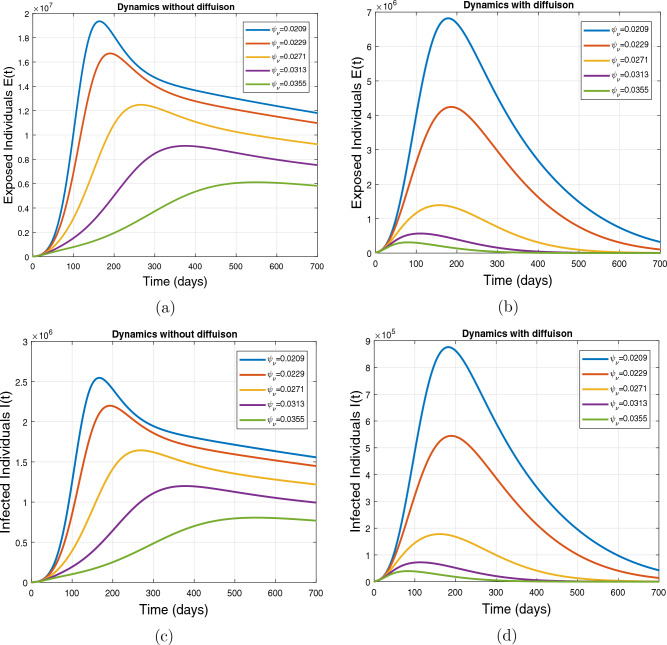
Impact of vaccination rate $\psi _{\nu }$, with and without diffusion at $x=0.0$ on exposed shown in (**a**,**b**) and on infected individuals shown in subplots (**c**,**d**).

**Figure 8:**
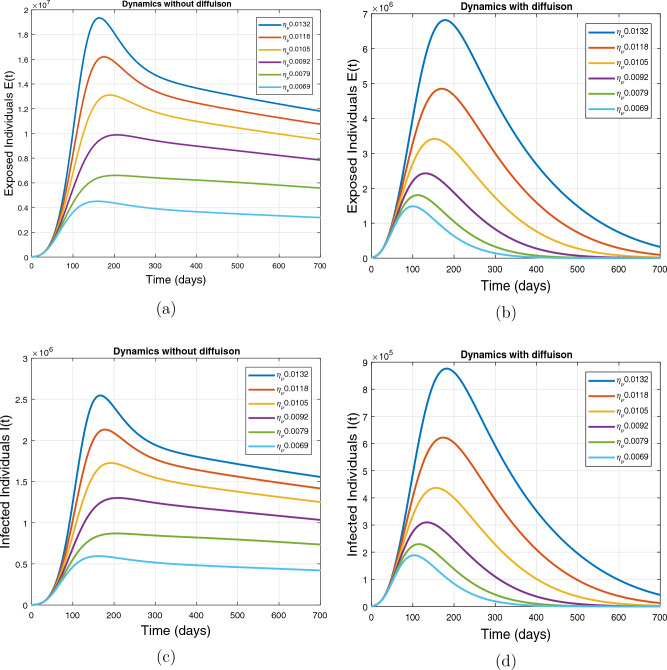
Impact of vaccine waning rate $\eta _{\nu }$ at $x=0.0$. The subplots (**a**,**b**) illustrate the dynamics of exposed individuals with and without diffusion respectively, and the subplots (**c**,**d**) present the respective profile of infected individuals with and without diffusion.

This subsection presents the simulation results describing the time evolutionary trajectories of the model (1) with and without diffusion at $x=0$ using FDOSM. Moreover, this section illustrates the role of various parameters including $\beta$, $\kappa$, $\psi _{\nu }$ and $\eta _{\nu }$ with diffusive and non-diffusive cases. These parameters correspond to the implementation of various pharmaceutical and non-pharmaceutical interventions such as social distancing measures and vaccination etc. These results are demonstrated in Figs. 5, 6, 7 and 8. Figure 5 illustrates the impact of control measure $\beta$ (the effective contact rate) on the exposed and infectious population. The dynamics are studied in both diffusive and non-diffusive cases as shown in Fig. 5a–d. The profiles of the exposed and infected populations are obtained for the baseline value $\beta = 0.4710$ given in Table 1 and it is decreased by $10\%, 20\%$ and $30\%$. It is observed that a reasonable reduction is analyzed in the aforementioned population classes. Moreover, the lowest projected peak up to $97\%$ reduction in exposed and symptomatically infected individuals is noticed with $30\%$ decrease in social contacts in presence of diffusion. The detailed description is presented in Table 2. Further, we demonstrate the impact of parameter $\kappa$ (the relative transmissibility rate of infection due to exposed individuals) population dynamics in both diffusive and non-diffusive cases as shown in Fig. 6a–d. The graphical results are analyzed initially for the baseline value, i.e. $\kappa = 0.3378$ in case of diffusion and without diffusion. One can observe the difference between the projected peak value presented in Table 2. Furthermore, the value of $\kappa$ is reduced by $10\%, 20\%$, and $30\%$ respectively and as a result, $92\%$ decrease is observed with diffusion. Figure 6 describes the role of the implementation of vaccination control measure $\psi _{\nu }$ on the disease incidence. The simulation is performed for the baseline value of this parameter i.e., $\psi _{\nu } = 0.0209$. Then $\psi _{\nu }$ is enhanced to $10\%, 20\%, 30\%$ and $40\%$ to the baseline value. It is noticed that exposed and symptomatically infected individuals get decreases and with $40\%$ enhancement in vaccination rate, a $95\%$ decrease is observed in the case of diffusion. According to biological facts, vaccines contain anti-viral agents that are responsible for boots the immunity of suspects and it will probably increase the recovery rate. That’s why a significant reduction in the infected population is observed. Figure 8 depicts the role of vaccine waning rate $\eta _{\nu }$ on exposed and symptomatically infected individuals. The behavior is analyzed by decreasing $10\%, 20\%, 30\%$ and $40\%$ vaccine waning rate from the baseline value $\eta _{\nu } = 0.0132$. A significant reduction is analyzed with $40\%$ decrease in vaccine waning rate. The more effective result is observed in the case of diffusion.

**Table 2:** Projected peaks of infected individuals generated by diffusive and non-diffusive model (1) using finite difference operator-splitting method at $x=0.0$.

Parameters	With diffusion			Without diffusion		
	*E*	*I*	$\%$ Change	*E*	*I*	$\%$ Change
$\beta$ (baseline value)	$6.8196\times 10^{6}$	$8.7611\times 10^{5}$	–	$1.9346\times 10^{7}$	$2.5462\times 10^{6}$	–
$10\%$ reduction	$2.5539\times 10^{6}$	$3.2716\times 10^{5}$	$62\%$	$1.4992\times 10^{7}$	$1.9738\times 10^{6}$	$22\%$
$20\%$ reduction	$6.7711\times 10^{5}$	$8.6406\times 10^{4}$	$90\%$	$1.0742\times 10^{7}$	$1.4144\times 10^{6}$	$44\%$
$30\%$ reduction	$2.0621\times 10^{5}$	$2.6248\times 10^{4}$	$97\%$	$6.1323\times 10^{6}$	$8.0755\times 10^{5}$	$68\%$
$\kappa$ (baseline value)	$6.8196\times 10^{6}$	$8.7611\times 10^{5}$	–	$1.9346\times 10^{7}$	$2.5462\times 10^{6}$	–
$10\%$ reduction	$3.5116\times 10^{6}$	$4.5033\times 10^{5}$	$48\%$	$1.6220\times 10^{7}$	$2.1353\times 10^{6}$	$16\%$
$20\%$ reduction	$1.3908\times 10^{6}$	$1.7791\times 10^{5}$	$79\%$	$1.3189\times 10^{7}$	$1.3291\times 10^{6}$	$31\%$
$30\%$ reduction	$5.1313\times 10^{5}$	$6.5465\times 10^{4}$	$92\%$	$1.0093\times 10^{7}$	$1.3291\times 10^{6}$	$47\%$
$\psi _{\nu }$ (baseline value)	$6.8196\times 10^{6}$	$8.7611\times 10^{5}$	–	$1.9346\times 10^{7}$	$2.5462\times 10^{6}$	–
$10\%$ enhancement	$4.2456\times 10^{6}$	$5.4462\times 10^{5}$	$37\%$	$1.6707\times 10^{7}$	$2.1992\times 10^{6}$	$14\%$
$20\%$ enhancement	$1.3910\times 10^{6}$	$1.7777\times 10^{5}$	$80\%$	$1.2483\times 10^{7}$	$1.6436\times 10^{6}$	$21\%$
$30\%$ enhancement	$5.6791\times 10^{5}$	$7.2340\times 10^{4}$	$92\%$	$9.1069\times 10^{6}$	$1.1992\times 10^{6}$	$53\%$
$40\%$ enhancement	$3.1110\times 10^{5}$	$3.9563\times 10^{4}$	$95\%$	$6.1171\times 10^{6}$	$8.0555\times 10^{5}$	$68\%$
$\eta _{\nu }$ (baseline value)	$6.8196\times 10^{6}$	$8.7611\times 10^{5}$	–	$1.9346\times 10^{7}$	$2.5462\times 10^{6}$	–
$10\%$ reduction	$4.8522\times 10^{6}$	$6.2205\times 10^{5}$	$28\%$	$1.6194\times 10^{7}$	$2.1318\times 10^{6}$	$16\%$
$20\%$ reduction	$3.4159\times 10^{6}$	$4.3681\times 10^{5}$	$50\%$	$1.3109\times 10^{7}$	$1.7259\times 10^{6}$	$32\%$
$30\%$ reduction	$2.4291\times 10^{6}$	$3.0970\times 10^{5}$	$64\%$	$9.8839\times 10^{6}$	$1.3015\times 10^{6}$	$48\%$
$40\%$ reduction	$1.8046\times 10^{6}$	$2.2955\times 10^{5}$	$73\%$	$6.6067\times 10^{6}$	$8.6998\times 10^{5}$	$66\%$
$50\%$ reduction	$1.4075\times 10^{6}$	$1.7869\times 10^{5}$	$79\%$	$4.0459\times 10^{6}$	$5.3265\times 10^{5}$	$79\%$

#### Simulation based on nonuniform initial condition at $x=1.0$

**Figure 9:**
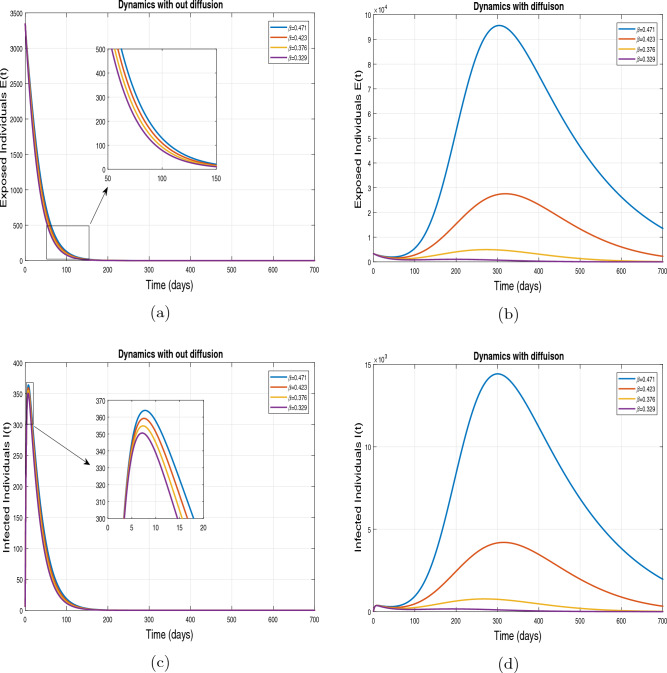
Effect of variation in $\beta$ at $x=1.0$ using FDOSM. The subplots (**a**,**b**) illustrate the dynamics of exposed individuals with and without diffusion respectively while the subplots (**c**,**d**) present the respective profile of infected individuals with and without diffusion.

The current section presents the dynamics of exposed and infected individuals based on nonuniform initial conditions (4) at $x=1.0$ using FDOSM. The simulation is performed for the model in diffusive and non-diffusive cases. The dynamics of exposed and infected populations with and without diffusion under the variation in $\beta$ is examined in Fig. 9. According to the initial conditions profiles given by Fig. 4, the population concentration is low at $x=1.0$. Therefore, in the absence of diffusion, the exposed and infected individuals are decreasing exponentially by decreasing the control measure $\beta$. On the other hand, when diffusion is involved the exposed and infected individuals rise for the baseline value $\beta = 0.4710$. Moreover, with the reduction in $\beta$ by $10\%, 20\%$ and $30\%$ to the baseline value, a reasonable reduction is observed in exposed and infected individuals. The projected peak values, in this case, are presented in Table 3.

**Table 3:** Projected numerical values of infected population observed by diffusive and non-diffusive model (1) using a finite difference operator-splitting method at $x=1.0$.

Parameters	With diffusion			Without diffusion		
	*E*	*I*	$\%$ Change	*E*	*I*	$\%$ Change
$\beta$ (baseline value)	$9.5615\times 10^{4}$	$1.4426\times 10^{4}$	–	$3.3511\times 10^{3}$	363.9654	–
$10\%$ reduction	$2.7555\times 10^{3}$	$4.1997\times 10^{3}$	$71\%$	$3.3099\times 10^{3}$	359.2260	$1.3\%$
$20\%$ reduction	$4.9569\times 10^{3}$	769.8151	$95\%$	$3.2609\times 10^{3}$	354.8060	$2.5\%$
$30\%$ reduction	$3.3508\times 10^{3}$	365.6436	$97\%$	$3.2309\times 10^{3}$	350.5837	$3.5\%$
$\kappa$ (baseline value)	$9.5615\times 10^{4}$	$1.4426\times 10^{4}$	–	$3.3511\times 10^{3}$	363.9654	–
$10\%$ reduction	$4.1933\times 10^{4}$	$6.3631\times 10^{3}$	$56\%$	$3.3159\times 10^{3}$	360.1763	$1.04\%$
$20\%$ reduction	$1.3141\times 10^{4}$	$2.0144\times 10^{3}$	$86\%$	$3.2829\times 10^{3}$	356.5208	$2.04\%$
$30\%$ reduction	$3.6340\times 10^{3}$	565.4072	$96\%$	$3.2499\times 10^{3}$	352.9999	$3.01\%$
$\psi _{\nu }$ (baseline value)	$9.5615\times 10^{4}$	$1.4426\times 10^{4}$	–	$3.3511\times 10^{3}$	363.9654	–
$10\%$ enhancement	$5.2369\times 10^{4}$	$7.9344\times 10^{3}$	$45\%$	$3.3487\times 10^{3}$	363.6771	$0.07\%$
$20\%$ enhancement	$1.2285\times 10^{4}$	$1.8868\times 10^{3}$	$87\%$	$3.3442\times 10^{3}$	363.0871	$0.24\%$
$30\%$ enhancement	$3.5773\times 10^{3}$	558.6458	$96\%$	$3.3379\times 10^{3}$	362.5168	$0.39\%$
$40\%$ enhancement	$3.5140\times 10^{3}$	3.809405	$97\%$	$3.3330\times 10^{3}$	361.9651	$0.54\%$
$\eta _{\nu }$ (baseline value)	$9.5615\times 10^{4}$	$1.4426\times 10^{4}$	–	$3.3511\times 10^{3}$	363.9654	–
$10\%$ reduction	$5.5566\times 10^{4}$	$8.4536\times 10^{3}$	$41\%$	$3.35099\times 10^{3}$	363.9560	$0.0025\%$
$20\%$ reduction	$3.0701\times 10^{4}$	$4.7202\times 10^{3}$	$67\%$	$3.35092\times 10^{3}$	363.9472	$0.0050\%$
$30\%$ reduction	$1.6438\times 10^{4}$	$2.5617\times 10^{3}$	$82\%$	$3.35085\times 10^{3}$	363.9384	$0.0074\%$
$40\%$ reduction	$9.1734\times 10^{3}$	$1.4522\times 10^{3}$	$90\%$	$3.35079\times 10^{3}$	363.9296	$0.0098\%$
$50\%$ reduction	$6.2113\times 10^{3}$	995.6936	$93\%$	$3.35070\times 10^{3}$	363.9227	$0.0117\%$

**Figure 10:**
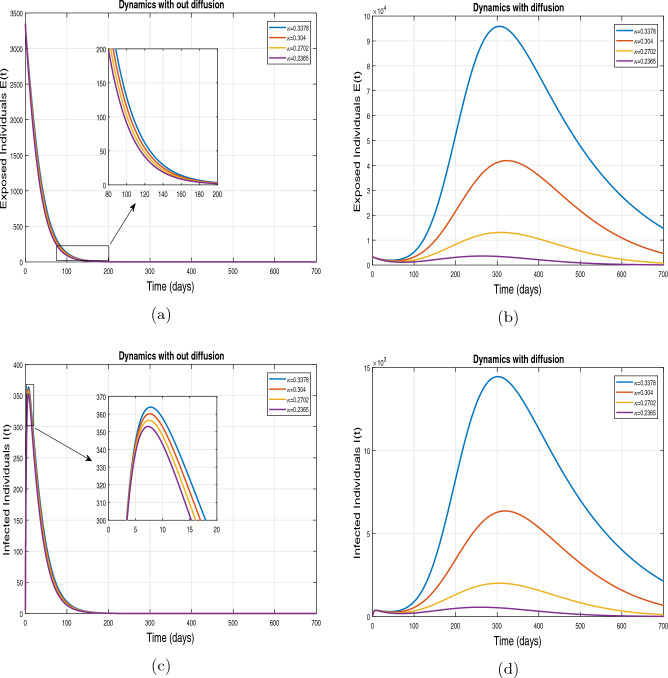
Impact of transmissibility rate $\kappa$ at $x=1.0$ using FDOSM. The subplots (**a**,**b**) illustrate the dynamics of exposed individuals with and without diffusion respectively, and the subplots (**c**,**d**) present the respective profile of infected individuals with and without diffusion.

Figure 10 depicts the time evolutionary trajectories obtained by using FDOSM for the control measure $\kappa$ with and without diffusion at $x=1.0$. According to Fig. 4, the exposed and infected individuals have low concentrations at $x=1.0$. Therefore, in the absence of diffusion, the number of exposed and infected individuals decreased exponentially and no significant reduction is observed by reducing $\kappa$. Moreover, in the case of diffusion i.e. the population moves from higher concentration to lower concentration, the exposed and infected population increased initially for the baseline value at $x=1.0$. Further, the simulation is performed for $10\%, 20\%$ and $30\%$ reduction in $\kappa$ where the lowest peak is noticed for exposed and infected individuals with $30\%$ reduction in the value of $\kappa$ as shown in Table 3.

**Figure 11:**
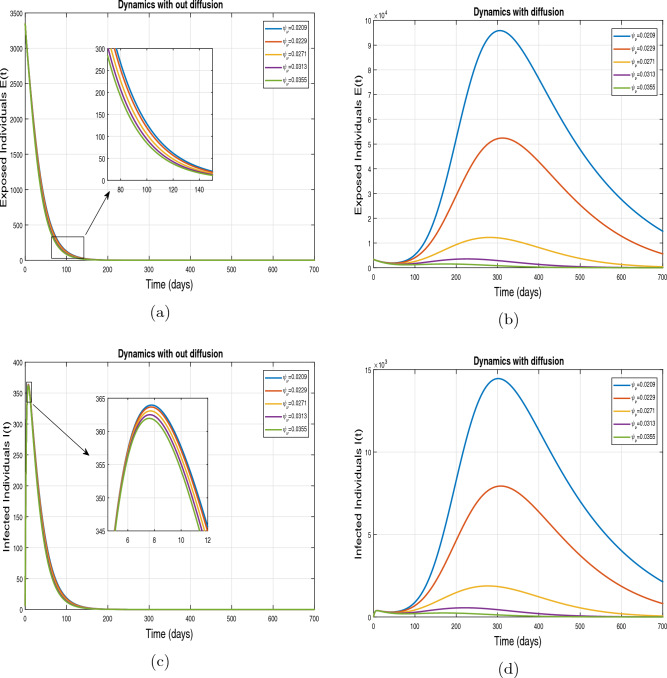
Impact of vaccination rate $\psi _{\nu }$ at $x=1.0$ using FDOSM. The subplots (**a**,**b**) illustrate the dynamics of exposed individuals with and without diffusion respectively while the subplots (**c**,**d**) present the respective profile of infected individuals with and without diffusion.

One of the most important control interventions against the COVID-19 pandemic is proper vaccination. The impact parameter describing the vaccination rate $\psi _{\nu }$ on the exposed and infected population dynamics at spatial point $x=1.0$ with and without diffusion is shown in Fig. 11. The evolutionary curve is obtained initially for the baseline value of $\psi _{\nu }$ i.e., 0.0209. Furthermore, the vaccination rate is enhanced by $10\%, 20\%, 30\%$ and $40\%$ to the actual value in order to analyze the dynamics of the infected population. According to initial condition (4) the population concentration at $x=1.0$ is low. As a result, if diffusion is not considered i.e., there is no spatial movement of the population, the exposed and infected population is decreased exponentially and enhancing the vaccination rate has no reasonable impact on the reduction of infection. On another hand, in the case of diffusion, as the population moves from higher concentration to lower concentration, the number of infected individuals initially increases for the baseline value of $\psi _{\nu }$. By enhancing the vaccination one can observe a significant reduction in the infected population. Moreover, the lowest peak is observed in the case of $40\%$ reduction in $\psi _{\nu }$ with diffusion.

**Figure 12:**
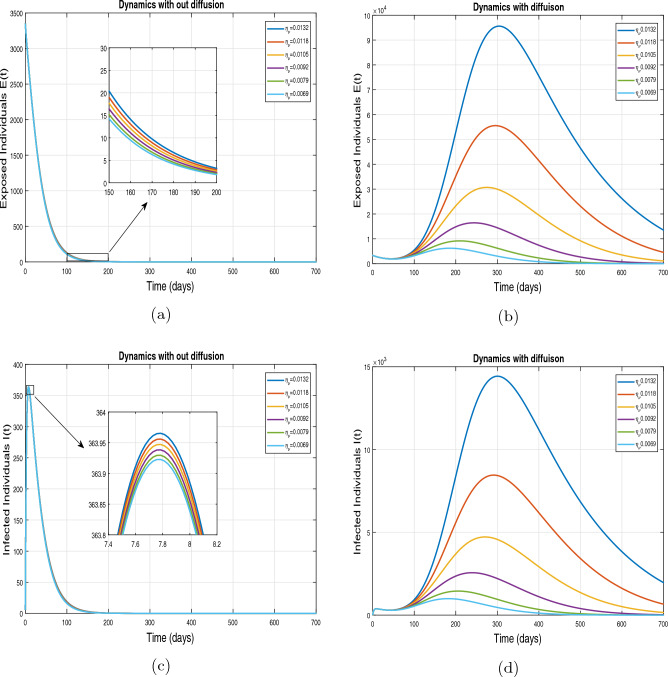
Impact of vaccine waning rate $\eta _{\nu }$ at $x=1.0$ using FDOSM. The subplots (**a**,**b**) illustrate the dynamics of exposed individuals with and without diffusion respectively and the subplots (**c**,**d**) present the respective profiles of infected individuals with and without diffusion.

The impact of vaccine waning rate $\eta _{\nu }$ on the respective population dynamics is described in Fig. 12 with and without diffusion at $x=1.0$. The behavior is observed for the baseline value 0.0132 of $\eta _{\nu }$ and then it is reduced by $10\%, 20\%, 30\%, 40\%$, $50\%$. From the evolutionary curve obtained by the FDOSM scheme, it is observed that in the absence of diffusion, the number of infected and exposed individuals reduces exponentially due to the low concentration of population at $x=1.0$. In the case of diffusion, the number of infected individuals increases for baseline value of $\eta _{\nu }$ initially, while decreasing with a small vaccine waning rate. That is smaller the vaccine waning rate, the lower will be the chances of reinfection. Thus, the individuals with minimum loss of immunity diffuses will have low chances of reinfection. Therefore, the number of infected individuals decreases.

### Simulation with mesh-free operator-splitting method

**Figure 13:**
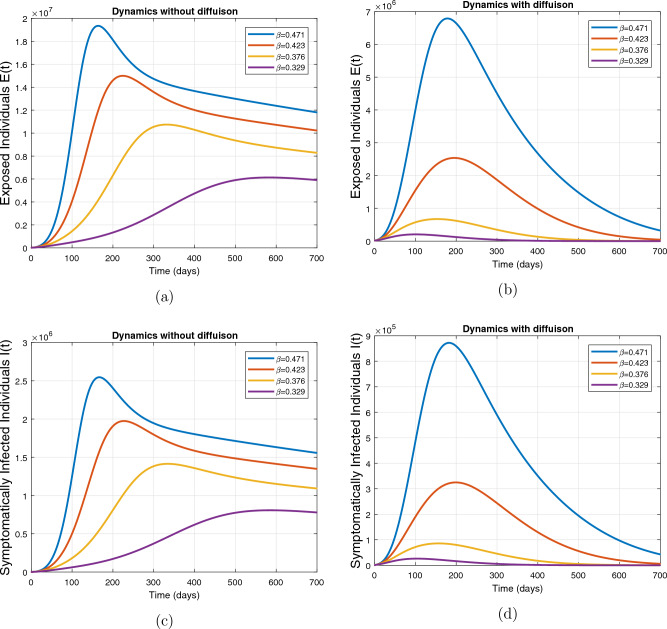
Impact of effective contact rate $\beta$, at $x=0.0$ using RBF. The subplots (**a**,**b**) illustrate the dynamics of exposed individuals with and without diffusion while the subplots (**c**,**d**) present the respective profile of infected individuals with and without diffusion.

**Figure 14:**
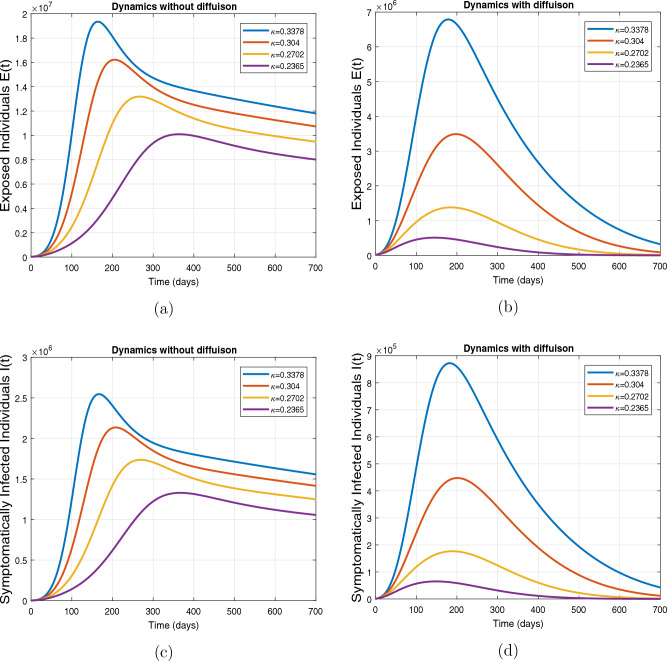
Impact of transmissibility rate $\kappa$ at $x=0.0$ using RBF. The subplots (**a**,**b**) illustrate the dynamics of exposed individuals with and without diffusion, and the subplots (**c**,**d**) demonstrate the respective profiles of infected individuals with and without diffusion.

**Figure 15:**
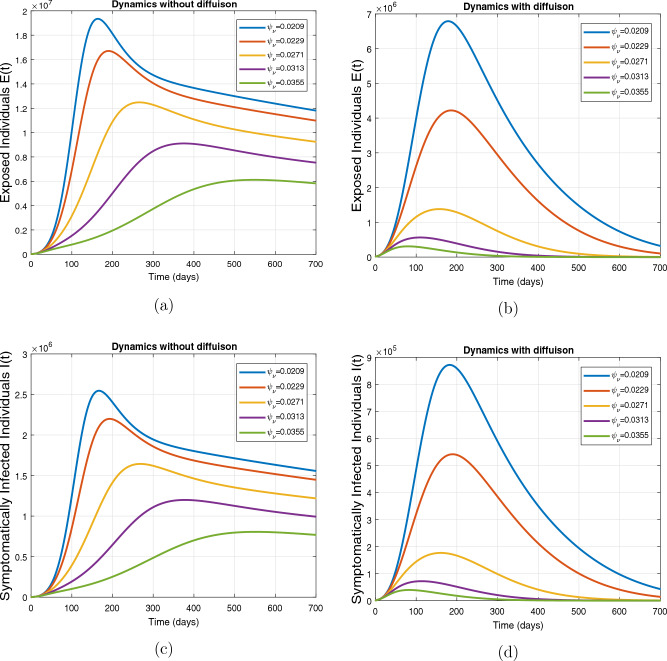
Impact of vaccination rate $\psi _{\nu }$, at $x=0$ using RBF. The subplots (**a**,**b**) illustrate the dynamics of exposed individuals with and without diffusion while the subplots (**c**,**d**) show the respective profiles of infected individuals with and without diffusion.

**Figure 16:**
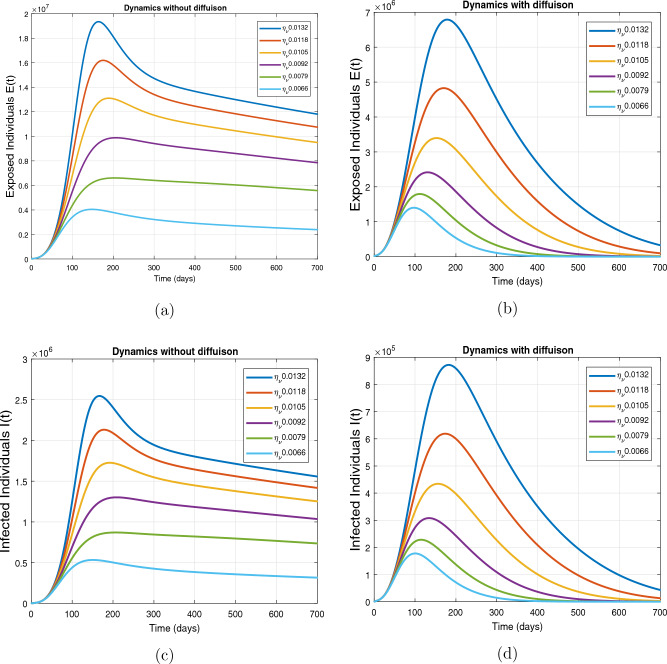
Impact of vaccine waning rate $\psi _{\nu }$ at $x=0$ using RBF. The subplots (**a**,**b**) depict the dynamics of exposed individuals with and without diffusion and the subplots (**c**,**d**) illustrate the respective profiles of infected individuals with and without diffusion.

**Figure 17:**
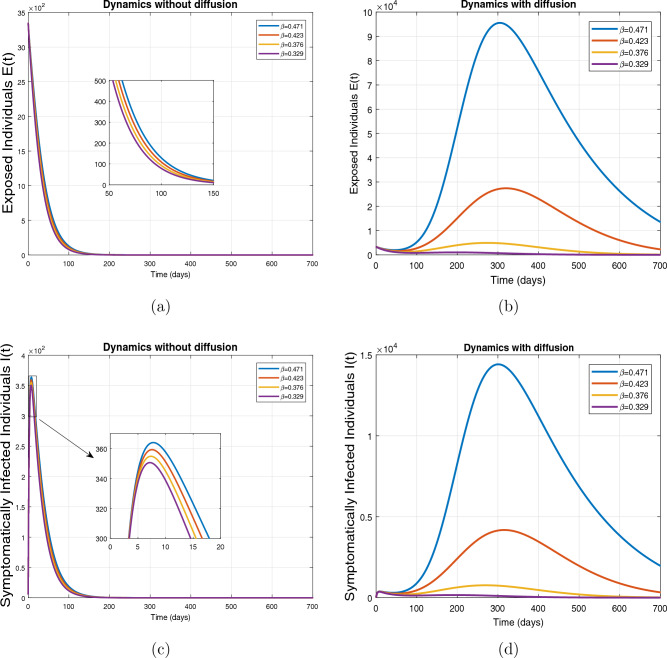
Impact of effective contact rate $\beta$ at $x=1.0$ using RBF where the subplots (**a**,**b**) illustrate the dynamics of exposed individuals with and without diffusion and the subplots (**c**,**d**) show the respective profile of infected individuals with and without diffusion.

**Figure 18:**
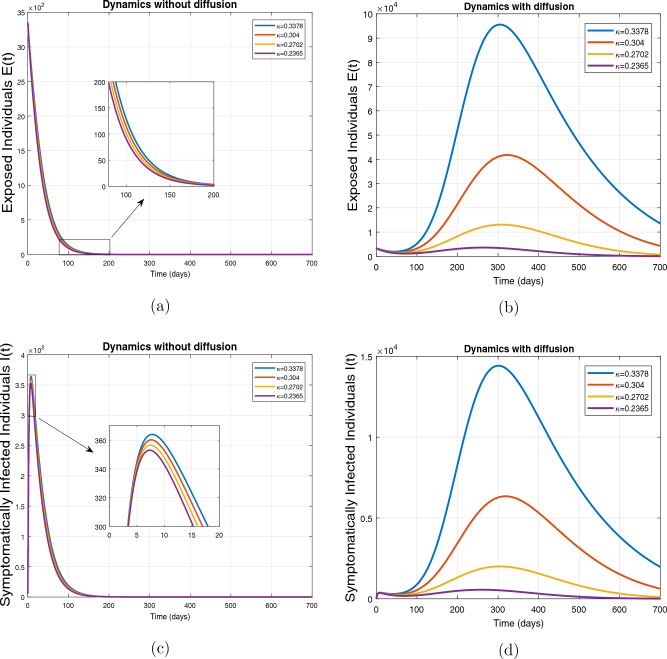
Impact of transmissibility rate $\kappa$ at $x=1.0$ using RBF. The subplots (**a**,**b**) illustrate the dynamics of exposed individuals with and without diffusion and the subplots (**c**,**d**) analyze the respective profiles of infected individuals with and without diffusion.

**Figure 19:**
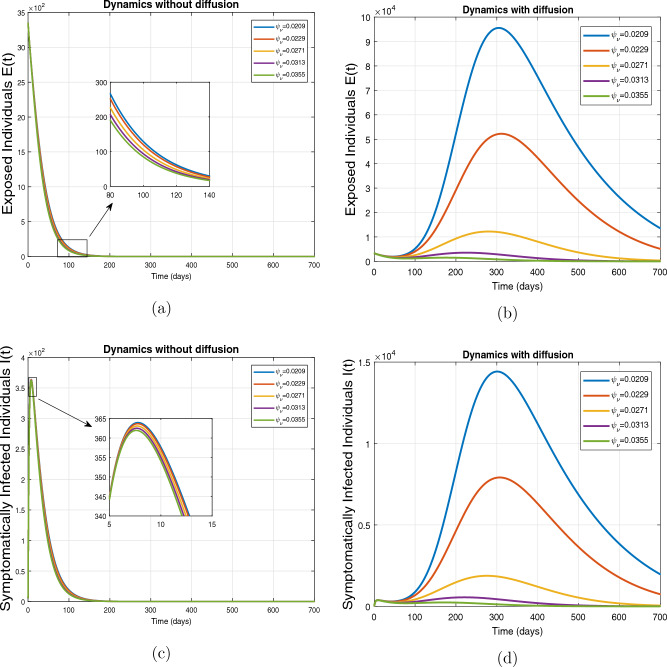
Impact of vaccination rate $\psi _{\nu }$ at $x=1.0$ using RBF. The subplots (**a**,**b**) demonstrate the dynamics of exposed individuals with and without diffusion and the subplots (**c**,**d**) illustrate the respective profiles of infected individuals with and without diffusion.

**Figure 20:**
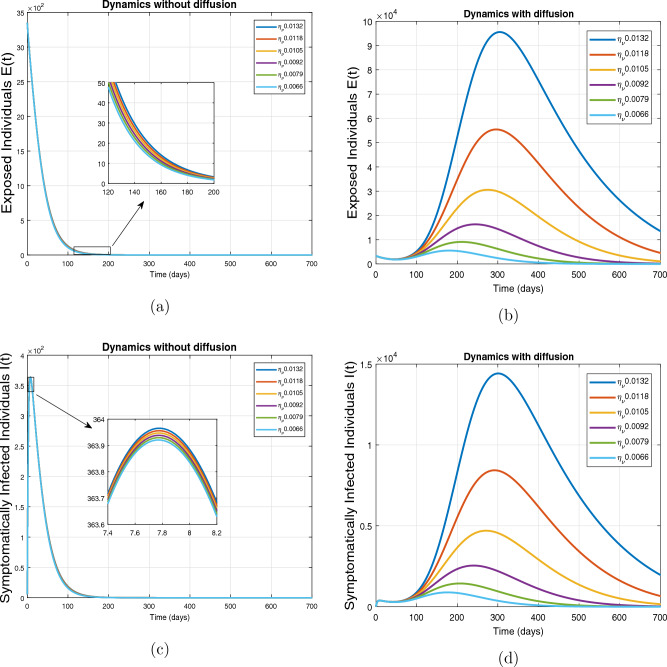
Impact of vaccination waning rate $\eta _{\nu }$ at $x=1.0$ using RBF. The subplots (**a**,**b**) demonstrate the dynamics of exposed individuals with and without diffusion and the subplots (**c**,**d**) illustrate the respective profiles of infected individuals with and without diffusion.

In this section, we describe the numerical simulation of the proposed spatio-temporal COVID-19 vaccine model (1) using a mesh-free operator-splitting iterative scheme based on multi-quadratic radial basis function for the time period of 700 days. Similar to the previous section, the simulation is performed at $x=0.0$ and $x=1.0$. The time step remains the same as in the previous approach, i.e., $\Delta t = 0.028$, while the spatial step is $\Delta x = 0.200$, corresponding to ${{\textbf {N}}} = 20$ spatial points. The detailed interpretation of the different control measures as discussed in the previous section is demonstrated in Figs. 13, 14, 15, 16, 17, 18, 19 and 20.

#### Simulation based on initial condition (4) at $x=0.0$

The graphical solution of exposed and infected classes at $x=0.0$ are obtained utilizing the mesh-free operator-splitting method and presented in Figs. 13, 14, 15 and 16. Moreover, the dynamics are analyzed in respective cases with and without diffusion for the nonuniform initial condition (4). It is noticed that the graphical results obtained by using a mesh-free operator-splitting iterative scheme show almost similar behavior at $x=0.0$ as acquired from finite difference operator-splitting technique can be observed in Figs. 5, 6, 7 and 8. A comparative analysis of the projected peaks and relative percentage difference of both schemes from the baseline value at $x=0.0$ and with diffusion is presented in Table 4.

It is noticed that the results obtained from both numerical techniques are similar. But the benefit of using mesh-free operator-splitting based on multi-quadratic radial basis function is that this approach is a unique distinction as it works on scattered data and does not require underlying meshes or structured nodes. This approach is easily extendable to higher dimensional reaction–diffusion models. Moreover, it produces a better result on a low number of collocation points as compared to the finite-difference operator splitting scheme.

**Table 4:** Comparative projected numerical values of infected individuals obtained by diffusive model (1) using FDOSM and RBF method at $x=0.0$.

Parameters	With diffusion using FDOSM			With diffusion using MOSM		
	*E*	*I*	$\%$ Change	*E*	*I*	$\%$ Change
$\beta$ (baseline value)	$6.8196\times 10^{6}$	$8.7611\times 10^{5}$	–	$6.7896\times 10^{6}$	$8.7222\times 10^{5}$	–
$10\%$ reduction	$2.5539\times 10^{6}$	$3.2716\times 10^{5}$	$62.5\%$	$2.5365\times 10^{6}$	$3.2492\times 10^{5}$	$62.3\%$
$20\%$ reduction	$6.7711\times 10^{5}$	$8.6406\times 10^{4}$	$90.0\%$	$6.7254\times 10^{5}$	$8.5821\times 10^{4}$	$90.1\%$
$30\%$ reduction	$2.0621\times 10^{5}$	$2.6248\times 10^{4}$	$96.9\%$	$2.0523\times 10^{5}$	$2.6121\times 10^{4}$	$96.9\%$
$\kappa$ (baseline value)	$6.8196\times 10^{6}$	$8.7611\times 10^{5}$	–	$6.7898\times 10^{6}$	$8.7225\times 10^{5}$	–
$10\%$ reduction	$3.5116\times 10^{6}$	$4.5033\times 10^{5}$	$48.6\%$	$3.4898\times 10^{6}$	$4.4752\times 10^{5}$	$48.5\%$
$20\%$ reduction	$1.3908\times 10^{6}$	$1.7791\times 10^{5}$	$79.7\%$	$1.3807\times 10^{6}$	$1.7661\times 10^{5}$	$79.6\%$
$30\%$ reduction	$5.1313\times 10^{5}$	$6.5465\times 10^{4}$	$92.5\%$	$5.0984\times 10^{6}$	$6.5043\times 10^{4}$	$92.4\%$
$\psi _{\nu }$ (baseline value)	$6.8196\times 10^{6}$	$8.7611\times 10^{5}$	–	$6.7898\times 10^{6}$	$8.7225\times 10^{5}$	–
$10\%$ enhancement	$4.2456\times 10^{6}$	$5.4462\times 10^{5}$	$37.8\%$	$4.2217\times 10^{6}$	$5.4154\times 10^{5}$	$37.9\%$
$20\%$ enhancement	$1.3910\times 10^{6}$	$1.7777\times 10^{5}$	$79.6\%$	$1.3818\times 10^{6}$	$1.7656\times 10^{5}$	$79.7\%$
$30\%$ enhancement	$5.6791\times 10^{5}$	$7.2340\times 10^{4}$	$91.6\%$	$5.6483\times 10^{6}$	$7.1943\times 10^{4}$	$91.7\%$
$40\%$ enhancement	$3.1110\times 10^{5}$	$3.9563\times 10^{4}$	$95.4\%$	$3.0981\times 10^{6}$	$3.9395\times 10^{4}$	$94.5\%$
$\eta _{\nu }$ (baseline value)	$6.8196\times 10^{6}$	$8.7611\times 10^{5}$	–	$6.7898\times 10^{6}$	$8.7225\times 10^{5}$	–
$10\%$ reduction	$4.8522\times 10^{6}$	$6.2205\times 10^{5}$	$28.9\%$	$4.8271\times 10^{6}$	$6.1882\times 10^{5}$	$29.2\%$
$20\%$ reduction	$3.4159\times 10^{6}$	$4.3681\times 10^{5}$	$50.1\%$	$3.3966\times 10^{6}$	$4.3432\times 10^{5}$	$50.4\%$
$30\%$ reduction	$2.4291\times 10^{6}$	$3.0970\times 10^{5}$	$64.6\%$	$2.4152\times 10^{6}$	$3.0797\times 10^{5}$	$64.8\%$
$40\%$ reduction	$1.8046\times 10^{6}$	$2.2955\times 10^{5}$	$73.6\%$	$1.7947\times 10^{6}$	$2.2829\times 10^{5}$	$73.7\%$
$50\%$ reduction	$1.4075\times 10^{6}$	$1.7869\times 10^{5}$	$79.6\%$	$1.4503\times 10^{6}$	$1.7776\times 10^{5}$	$79.7\%$

#### Simulation based on initial condition (4) at $x=1.0$

**Figure 21:**
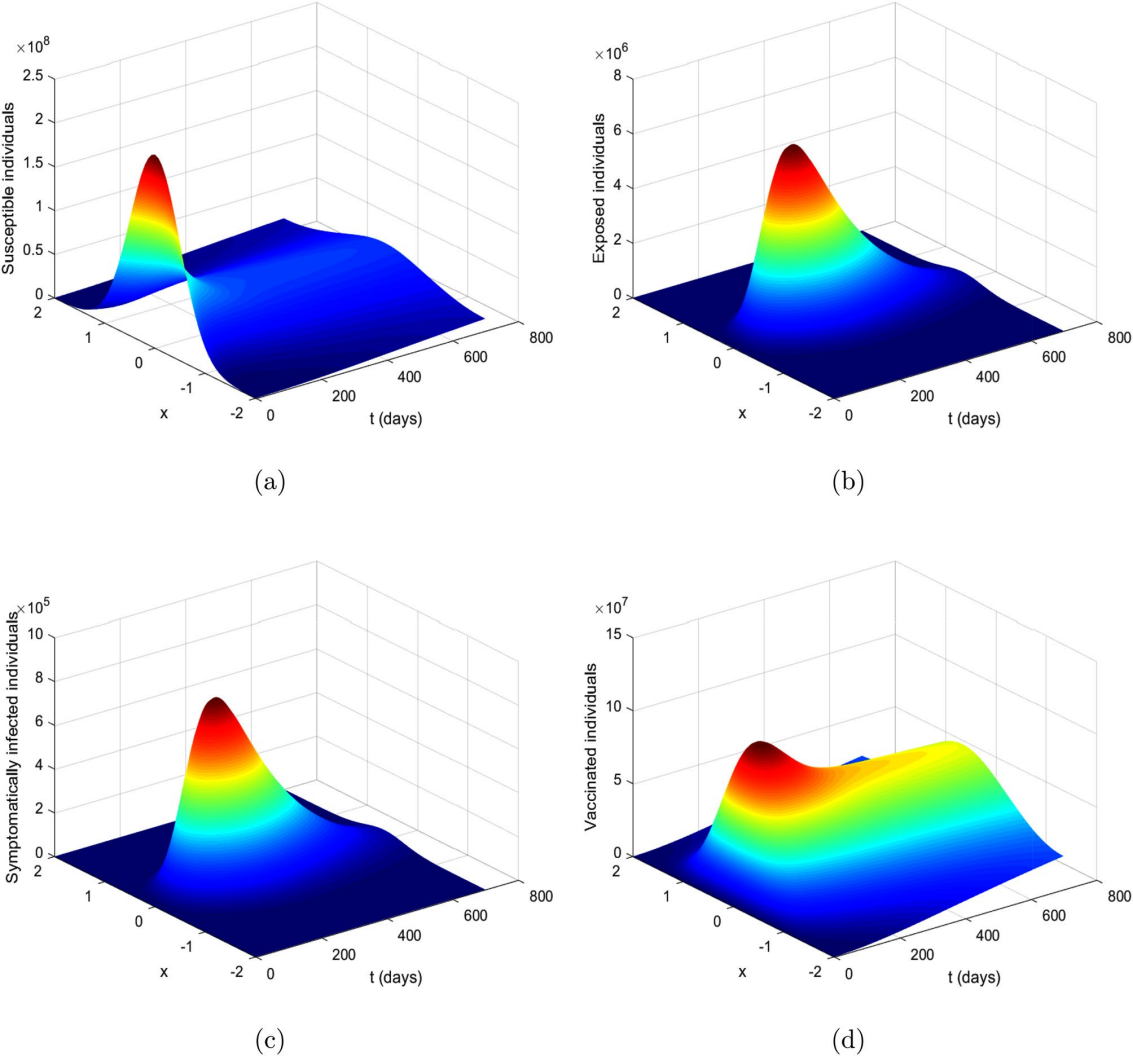
Mesh plots of (**a**) susceptible, (**b**) exposed, (**c**) symptomatically infected and (**d**) vaccinated individuals with diffusion.

**Figure 22:**
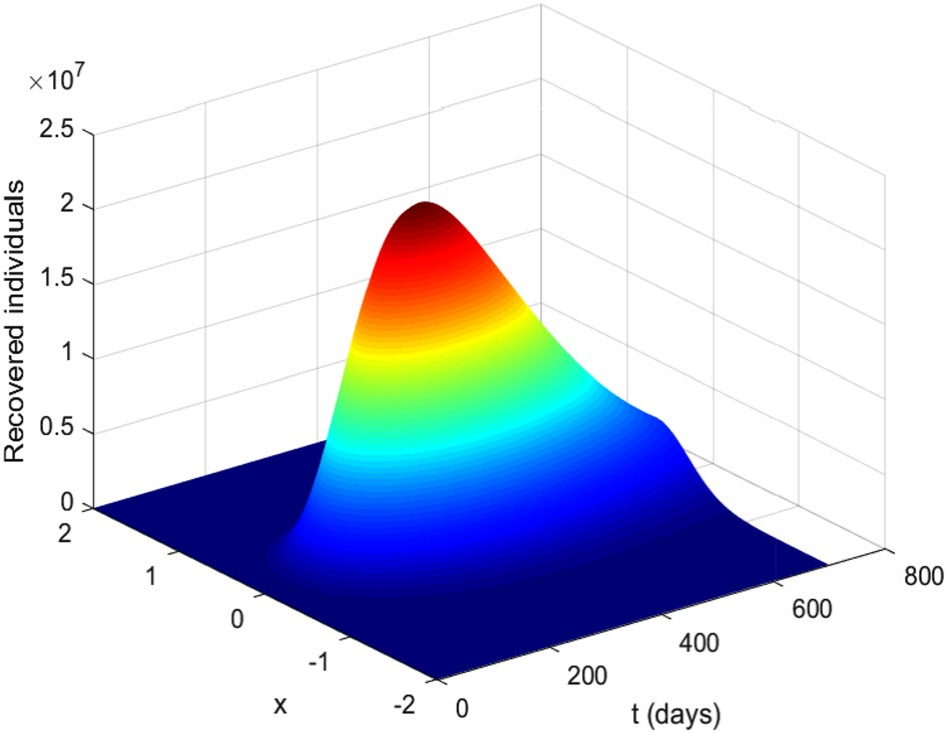
Mesh plot of recovered individuals with diffusion.

Finally, in Figs. 21 and 22, we present mesh plots of model (1) to study the combined spatial and temporal dynamics of different populations on the entire time and spatial points in $[-2, 2]$. The mesh plots associated with the proposed operator-splitting meshless numerical scheme demonstrate a consistent behavior. It is evident that the scheme effectively maintains the positivity property of the solution, ensuring that the solution remains positive for all $t>0$ and spatial points within the defined domain $[-2, 2]$. Moreover, the solution converges toward the steady states. In addition, according to the initial population distribution given by (4) concentration of the population is higher at the center of the spatial domain for the time period 0–10 days and then gradually diffuses in $[-2, 2]$ with time. Therefore, the exposed population is increasing due to the spatial movement of susceptible and as a result of interaction with infected and exposed individuals. Therefore, the population of infected as well as the vaccinated compartments increased.

## Conclusion

In the present study, a spatio-temporal COVID-19 vaccine model is formulated and analyzed qualitatively and numerically. The aim of this work is to analyze the impact of diffusion as well as some control interventions including social distancing (by reducing effective contacts), vaccination rate and vaccine waning rate on the dynamics of the infected population in a spatially heterogeneous environment. Initially, the spatio-temporal COVID-19 model is analyzed qualitatively in detail. The basic mathematical analysis including the existence, uniqueness, boundedness and positivity of the solution is presented. The local and global stability of the spatio-temporal model at the steady sates is shown using well-known techniques. Moreover, the model is solved numerically based on uniform and non-uniform initial conditions with two different numerical schemes named: finite difference operator-splitting and mesh-free operator-splitting based on multi-quadratic radial basis functions. A detailed simulation is presented using both numerical schemes for diffusive and non-diffusive cases at two different spatial points $x=0$ and $x=1$. Two different spatial points are chosen due to the fact that $x=0$ represents the highly populated area as compared to $x=1$. The simulation results concluded that based on the initial conditions (3) and (4) at different spatial locations, the diffusion effect will play a significant role in curtailing infection transmission in a heterogeneous environment with concentrated regions. Further, it is observed that reducing $\beta$ to 30% in the case of diffusion, the infection will be reduced to 97% while enhancing vaccination rate to 40% a 95% decrease is observed in the infection incidence. The restriction of public gatherings during COVID-19 outbreak is important as the virus is airborne and easily transmit with low social distancing. Thus, it is concluded that implementing the suggested control strategies with diffusion is beneficial and helps in the eradication of infection from the community.

## Data Availability

The data that support the findings of this study are available from the corresponding author upon reasonable request. Further, no experiments on humans and/or the use of human tissue samples involved in this study.
